# Optimization of hyphenated asymmetric flow field-flow fractionation for the analysis of silver nanoparticles in aqueous solutions

**DOI:** 10.1007/s00216-021-03647-3

**Published:** 2021-09-19

**Authors:** Felix Geißler, María Martínez-Cabanas, Pablo Lodeiro, Eric P. Achterberg

**Affiliations:** 1 Chemical Oceanography, Marine Biogeochemistry, GEOMAR Helmholtz Centre for Ocean Research Kiel, Kiel, Germany; 2 Department of Chemistry, University of Lleida – AGROTECNIO-CERCA Center, Rovira Roure 191, 25198 Lleida, Spain

**Keywords:** AgNP, Marine waters, AF4, Aggregation

## Abstract

The extensive use of silver nanoparticles (AgNPs) in consumer products, medicine, and industry leads to their release into the environment. Thus, a characterization of the concentration, size, fate, and toxicity of AgNPs under environmental conditions is required. In this study, we present the characterization and optimization of an asymmetric flow field-flow fractionation (AF4) system coupled with UV/Vis spectrophotometer and dynamic light scattering (DLS) detector as a powerful tool for the size separation and multi-parameter characterization of AgNPs in complex matrices. The hyphenated AF4-UV/Vis-DLS system was first characterized using individual injections of the different size fractions. We used electrostatically stabilized AgNPs of 20-, 50-, and 80-nm nominal diameters coated with lipoic acid. We investigated the effect of applied cross-flows, carrier solutions, focus times, and quantity of injected particles on the nature of the AF4 fractograms and on the integrity of the AgNPs. Best size separation of a 1:1 mixture of 20- and 80-nm AgNPs was achieved using cross-flows of 0.5 and 0.7 mL/min with 1 mM NaCl and 0.05% v/v Mucasol as carrier solutions. We also researched the behavior of AgNPs in natural waters using the hyphenated AF4-UV/Vis-DLS system, under determined optimal conditions.

**Graphical abstract:**

**Figure:**
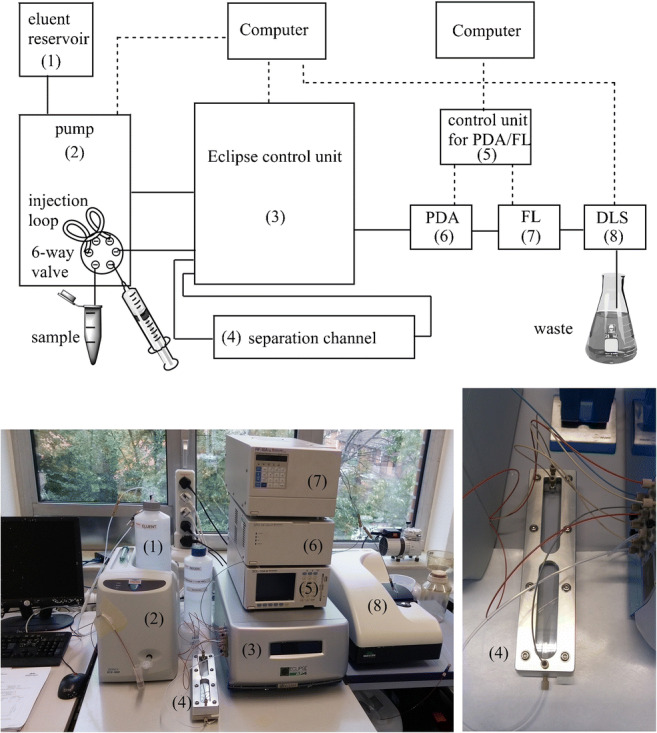
Schematic and photograph of the AF4 setup with numbered hardware devices. Dashed lines represent electrical connections; continuous lines represent fluidic connections. For a better overview, not all fluidic connections between pump/6-way valve (2) and the Eclipse AF4 device (3) are shown in the schematic. The fluorescence detector (FL (7)) was not used in the study presented herein.

**Supplementary Information:**

The online version contains supplementary material available at 10.1007/s00216-021-03647-3.

## Introduction

Nanoparticles are materials with nanometric size, typically below 100 nm. The small size of the nanoparticles, together with their large surface to volume ratio, leads to distinctive properties that make them different from the bulk material [1].

Silver nanoparticles (AgNPs) are one of the most widely used nanoparticles, with a large range of applications [2]. The unique optical properties of the AgNPs are well known and make them useful for biosensing and imaging applications [3–5]. The AgNPs can also be used in catalyzed reactions [6–8] and electronic applications [9–11]. Nevertheless, the most common and widely utilized properties of AgNPs are their antibacterial, antiviral, and biocidal abilities. These properties make AgNPs to be widely used in medical products, disinfectants, and food packing materials cosmetics [12], and as antiviral agents and drug delivery carriers for several human diseases like HIV-1, hepatitis, and even COVID-19 [13, 14]. The extensive production and consumption of AgNPs inevitably lead to increased exposure for humans and ecosystems. The AgNPs enter ecological systems as a consequence of leaching and recycling processes, through discharge in wastewater or atmospheric deposition [15, 16]. The European Union has reported that AgNPs are the second most abundant nanoparticles in surface waters with average concentrations around 1.5 ng·L^−1^ [1]. Very few studies were able to measure AgNPs in natural waters, with concentration levels depending on the studied locations. For example, AgNP levels between 2.0 and 8.6 ng L^−1^ were found in the river Isar (Germany) [17], while a recent study in Besós river basin (Spain) showed an average value of 640 pg L^−1^, one order of magnitude lower [18].

The AgNPs’ behavior and fate largely depend on their specific properties (e.g., capping agent, surface charge, particle diameter) and the characteristics of the surrounding medium (e.g., electrolyte composition, pH, ionic strength, presence of organic matter) [1, 19, 20]. Thus, the research of both the pristine NP characteristics and their modifications due to specific medium parameters is key to constrain potential toxic effects [21, 22]. In order to stablish the fate and toxicity of the AgNPs in the environment, it is therefore essential to develop methods and techniques to study their stability, distribution, and transport [16].

Field-flow fractionation (FFF) is one of the techniques with a higher potential to evaluate the behavior of the nanoparticles in aquatic environments. The FFF is a versatile fractionation method which allows the analytical separation and characterization of molecules and particles in a large range of sizes (from nm to μm) [23]. The FFF separation takes places in a thin ribbon-like channel and is based on the combination of the action of a carrier solution with a hyperbolic velocity profile, together with a physical field applied perpendicularly to this carrier liquid. Thus, the sample is carried downstream at different velocities and eluted from the channel with different retention times [24, 25]. In the last decades, multiple variations of FFF have been developed based on the type of separation force. The most versatile is the flow field-flow fractionation (FlFFF) that can be applied to compounds in the size range from 20 nm to 100 μm. This sub-technique uses a secondary flow (cross-flow) as field force. By the application of the cross-flow, the analytes are driven towards the channel’s permeable membrane, and the fractionation is achieved due to different diffusion coefficients. The FlFFF has mainly two possible setups: the symmetric FlFFF, where two independent flows are applied; and the asymmetric FlFFF or AF4, where the two flows (longitudinal and cross-flow) are produced by the same inlet [26]. The efficiency of the FlFFF technique in terms of size detection and quantification of manufactured AgNPs was evaluated by Cascio et al. [27], among other well-established methods, such as centrifugal liquid sedimentation or transmission electron microscopy (TEM) analyses.

Despite its multiple advantages, the use of FFF as a sorting tool for engineered nanoparticles in industrial processes is limited due to the inability to scale up the process. The implementation of new separation techniques like the zonal rotor centrifugation, based on the creation of density gradients [28], or the magnetic bearing-based high-speed centrifugation [29] opens the possibility to carry out the separation of different-sized nanoparticles in large-scale systems. Despite the promising results of these methods for the high-resolution separation of nanoparticle samples, there is a lack of information on their capability to be hyphenated to other instruments (like DLS, ICP, or UV/Vis), an essential feature to make easier the characterization of the separated fraction sizes.

The hyphenation of the non-destructive FFF system with various detectors is the main powerful advantage for the characterization of nanoparticle-containing samples. A method to achieve multidimensional information (e.g., hyphenated techniques) is highly desirable for the analysis of environmental samples, which is normally challenging due to complex sample matrices and low nanoparticle concentrations [30]. A number of studies have successfully evaluated the on-line hyphenation of the FlFFF to several detectors such as UV/Vis, fluorescence, ICP-MS, or dynamic light scattering (DLS) [31–36]. Marassi and co-workers demonstrated the viability of hollow-fiber flow field-flow fractionation hyphenated with multi-angle light scattering for the characterization of AgNPs in both pharmaceutical and medical products, and aqueous environments [37–39]. The FlFFF was successfully used to evaluate the size distribution in soils and water of natural colloids (like humic and fulvic acids) and their association to trace metals [40–44]. The effect of organic matter on the size and surface of PtNPs in artificial and natural waters was recently studied by Sánchez-Cachero and co-workers using AF4 hyphenated with ICP-MS [32]. The viability of AF4 for the separation and size characterization of ZnO nanoparticles in river and lake water was confirmed by Amde et al. [45]. Regarding the AgNPs, the use of FlFFF for their fractionation and characterization in freshwaters was reported by several authors. Kim and co-workers probed the efficiency of AF4 coupled with a liquid capillary cell for the size determination of natural AgNPs in groundwater [46]. Loosli et al. used a AF4-ICP-MS system for the characterization of natural and engineered AgNPs extracted from river waters [47, 48]. The characterization of AgNPs in river and lake waters by AF4 demonstrated the effect of natural organic matter on the stabilization of the nanoparticles [49]. Other studies showed the promising capacity of AF4 as a tool for analyzing the persistence and transformation of AgNPs in littoral lake mesocosms and wastewaters [50, 51]. More recently, Boughbina-Portolés et al. analyzed the stability of AgNPs in different water matrices by using an AF4-UV/VIS-DLS system [52]. Nevertheless, there is still a lack of FlFFF studies in marine and estuarine water samples, in terms of characterization, quantification, and stability of AgNPs derived from natural and anthropogenic sources, and more specifically the optimization of the selected AF4 parameters for AgNP separation in complex matrices.

In this work, an AF4 system was hyphenated with the UV/Vis and DLS detectors to characterize and investigate the behavior of AgNPs of 20, 50, and 80 nm coated with lipoic acid. The AgNP concentrations used in this study are in the order of milligrams per liter. The aggregation and dissolution of NPs are influenced by their concentration in solution; thus, caution is advised when extrapolating the results to very low AgNP concentrations (ng/L) of environmental relevance. Our aims were to investigate the effect of applied cross-flows, carrier solutions, focus times, and quantity of injected particles on the nature of the AF4 fractograms, recoveries, and on the behavior of the AgNPs. We also report the results of a case study assessing the aggregation of AgNPs in natural fjord waters under previously optimized AF4 conditions. Overall, we consider the provided information novel and relevant for the optimization of AF4 separation techniques with NPs.

Our final goal is to stablish a method for the characterization of the stability, size distribution, transport, and concentration of AgNPs in high ionic strength matrices such as estuarine and marine waters.

## Materials and methods

Spherically shaped AgNPs coated with lipoic acid (AgNP_LA) were purchased from nanoComposix (Prague, Czech Republic) in three different nominal sizes given by the supplier (20, 50, and 80 nm in diameter) as 1 g/L dispersions (BioPure™) in ultrapure water. These stock dispersions were diluted with ultrapure water obtained from a Milli-Q water purification system (Merck group), with a resistivity of 18.2 MΩ·cm and total organic carbon of <5 ppb, to 10 and 50 mg/L working dispersions. These working solutions were kept refrigerated at 5 °C in polypropylene vials covered from light. Before use, the working dispersions were treated for 2 min in an ultrasonic bath in order to eliminate any agglomerates.

The AF4 separation system used in this study is shown in the graphical abstract. Carrier and cleaning solutions were supplied to the separation system via a Dionex ICS-900 pump (Thermo Fisher Scientific, USA). The isocratic pump comprised a 6-way valve for fluidic control and an injection loop (Rheodyne) which was manually loaded with AgNP working dispersions using a syringe. Loops with an internal volume of 20 and 100 μL were used for the injection of 50 and 10 mg/L AgNP dispersions, respectively, equal to the injection of 1.0 μg of AgNPs. An Eclipse AF4 system (Wyatt Technology Europe GmbH, Germany) regulated all programmed steps of the separation including applied flow profiles, rates, directions, durations, and pressures. The Dionex pump and the Eclipse AF4 system were controlled via the software Chromeleon (version 6.80 SR13, Thermo Fisher Scientific, USA). Fluidic connection between pump and Eclipse AF4 and between Eclipse AF4 and separation channel was facilitated via polyether ether ketone (PEEK) tubing with inner diameters of 0.5 and 0.25 mm, respectively. The length of the separation channel was 29 cm with a height of 350 μm, defined by the dimensions of the used trapezoidal-shaped PEEK spacer. The separation membrane (Wyatt Technology Europe GmbH, Germany) was located between spacer and bottom ceramic frit and was made of polyether sulfone (PES) with a cutoff of 10 kDa. The membranes were replaced after ca. 50 consecutive runs. After replacement, at least five runs were performed in order to condition the new membrane with AgNPs. Silver nanoparticles were introduced from the injection loop onto the separation membrane using a flow rate of 0.2 mL/min. A flow rate of 0.5 mL/min was chosen for the detector and the channel. The separation protocol comprised six consecutive steps, each characterized by its individual duration and applied cross-flow rate (*V*_*x*_) as shown in the example of Table S1.

For the evaluation of the quality of the separation and the characterization of the AgNP fractions’ optical properties, UV/Vis spectra were recorded using a Shimadzu SPD-M10Avp photodiode array detector (PDA, graphical abstract) linked to the Eclipse AF4 device with 0.25-mm ID PEEK tubing and controlled with a SCL-10Avp unit via the LCsolution software 1.03 SP3 (Shimadzu). UV/Vis spectra were analyzed at the respective absorption maximum (*λ*_max_) of the individual AgNP size fractions, with *λ*_max_(*d* = 20 nm) = 399 nm, *λ*_max_(*d* = 50 nm) = 422 nm, and *λ*_max_(*d* = 80 nm) = 469 nm. Fractograms of a 1:1 mixture of 20- and 50-nm as well as 20- and 80-nm AgNPs were analyzed at wavelengths of 410 and 434 nm, respectively (average wavelength of the individual absorption maxima). For a characterization of the AgNP fractions in terms of particle size via dynamic light scattering (DLS), a ZetaSizer Nano-ZS (Malvern, UK; DLS (8) in graphical abstract) was linked to the Eclipse AF4 device with 0.25-mm ID PEEK tubing. The particle size was determined every 3 s using a quartz flow-through cell ZMV1008 (Hellma Analytics, Germany; 3 × 3 mm, 8.5-mm center height). In order to provide sufficient backpressure for the system (ca. 13 bar was required with a detector flow rate of 1 mL/min and no applied cross-flow according to the Eclipse AF4 handbook), the length of a 0.125-mm ID PEEK tubing at the sample outlet towards the waste was adjusted accordingly.

Three different carrier solutions were used for the AF4 experiments:

1. pH-adjusted ultrapure water (pH = 8, I = 0.02 mM); from now on “water pH 8”
2. Sodium chloride 1 mM (NaCl, reagent grade; Fisher Chemicals, UK) in ultrapure water (pH = 8)
3. Mucasol® 0.05% v/v (alkaline surfactant; Merz GmbH, Germany) in ultrapure water (pH = 10.5)

The pH of ultrapure water and 1 mM NaCl was adjusted with 1 M sodium hydroxide (NaOH, 98.5%; Acros Organics, Belgium). At the beginning of each experimental day, the system was rinsed with the respective carrier solution for at least 30 min. The first run with an injected sample was performed to ensure a saturation of the membrane and the acquired fractogram was not used in subsequent analyses. At the end of every measurement day, the whole system was flushed with a mixture of ultrapure water and ethanol (10% v/v EtOH; Merck, Germany) to avoid any crystallization in the tubing and separation channel. In order to remove any potential accumulations of AgNPs or silver chloride species inside the fluidic system and separation channel, the system was washed after ca. every 40–50 runs with an aqueous solution, which contained 0.5% v/v Mucasol and 1 mM nitric acid (65% p.a. HNO_3_; AppliCem, Germany).

For the characterization of the optical properties of the AgNPs independently from the AF4 system, a spectrophotometric system consisting of a DTMini-2-GS deuterium tungsten halogen light source (Ocean Optics, USA) and a USB4000 spectrophotometer (Ocean Optics, USA) was used. These devices were connected via optical fibers to the sample holder containing a 1 × 1 cm quartz cuvette (Agilent Technologies). The USB4000 spectrophotometer was operated in a wavelength range of 200 to 850 nm with the Ocean View Spectroscopy Software Version 1.4.1. For the acquisition of UV/Vis spectra, the AgNP stock dispersions were diluted to a mass concentration of 5 mg/L.

Size distributions as well as zeta-potentials of the AgNP batches were determined using the abovementioned ZetaSizer Nano-ZS equipment. The determination of the size distributions was performed with a mass concentration of 1 mg/L of the respective AgNPs in ultrapure water using disposable cuvettes (ZEN0040; Malver, UK). Zeta-potentials were determined with 2.5 mg/L AgNP dispersions in ultrapure water using folded capillary cells (DTS 1060; Malvern, UK). Electrophoretic mobility measurements were transformed to zeta-potentials using Henry’s equation under the Smoluchowski’s approximation using the software provided by the Malvern instrument [53, 54]. Aggregation experiments were conducted in a natural fjord water surface sample collected in Kiel Fjord (54.368° N, 10.195° E; southwest Baltic Sea, north Germany). A total of 1.9 mL fjord water was spiked with 0.1 mL of 50 mg/L AgNP_LA dispersions. The fjord water sample was characterized, and the main parameters measured are presented elsewhere [55]. The determined pH (7.7), salinity (17.19), and total organic carbon (277 μM) values are the expected for large estuarine-like systems, such as the Kiel Fjord.

In order to offer a complete approach for the analysis of AgNPs on the hyphenated AF4 system, we evaluated a large variety of settings: effect of carrier solution, effect of applied cross-flow, effect of focus time, recovery or quality of separation. The experiments were performed in single runs or duplicates. Table 1 summarizes all the experiments conducted with the hyphenated AF4 system.

**Table 1:** Summary of the hyphenated AF4 experimental design. Experiments were conducted in single runs or duplicates

Test	Sample	Elution conditions		
		Carrier solution	V_x_ (mL min^−1^)	Focus time (min)
**Effect of carrier solution and applied cross-flow**	Individual AgNP_LA fractions (20 nm, 50 nm, and 80 nm)	Water pH 8 0.05% v/v Mucasol 1 mM NaCl	0.0 0.2 0.5 1	10
**Effect of focus time**	50 nm AgNP_LA	Water pH 8	0.5	2 5 10
**Recovery**	20 nm AgNP_LA	Water pH 8 0.05% v/v Mucasol 1 mM NaCl	0.0 0.2 0.5 0.7 1	10
**Quality of separation**	Mixture 1:1 20 nm and 50 nm AgNP_LA	Water pH 8	0.5 1	10
	Mixture 1:1 20 nm and 80 nm AgNP_LA	Water pH 8 0.05% v/v Mucasol 1 mM NaCl	0.3 0.5 0.7	
**Separation of AgNPs in marine coastal waters**	Mixture 1:1 20 nm and 80 nm AgNP_LA spiked into fjord water	0.05% v/v Mucasol	0.5	10

## Results and discussion

### Characterization of AgNP batches

**Fig. 1:**
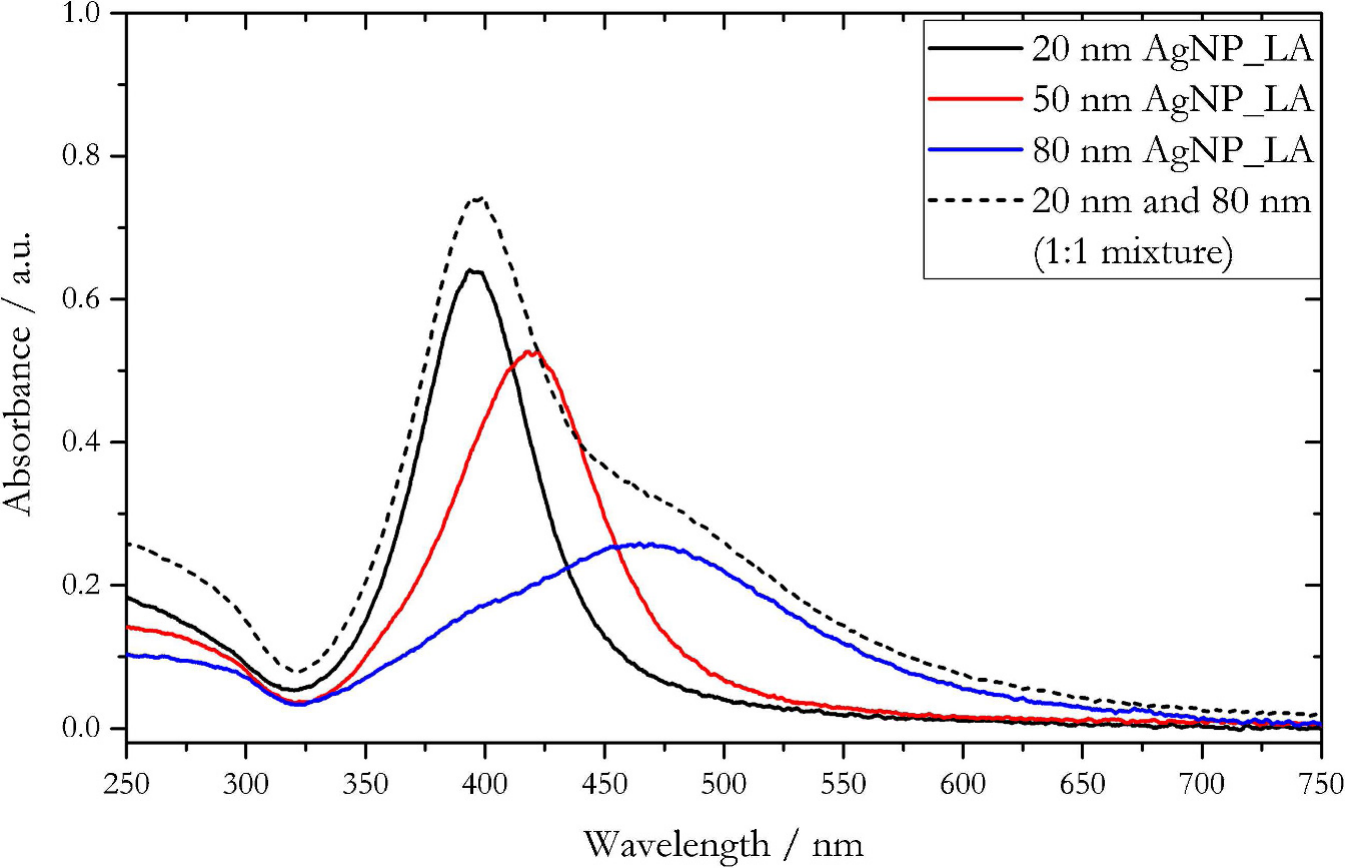
UV/Vis spectra of the individual AgNP_LA batches and of a 1:1 mixture of 20-nm and 80-nm particles. All spectra were acquired in ultrapure water

Prior to the conceptualization of any AF4 size separation experiment, the AgNPs coated with lipoic acid were carefully characterized in terms of size distribution, polydispersity, and zeta-potential (Table 2) as well as their optical properties (Fig. 1).

**Table 2:** Size characterization of AgNP_LA in batch and in AF4-UV/Vis-DLS system. Results obtained with the ZetaSizer Nano-ZS for the hydrodynamic diameter *d*_*H*_, polydispersity index *PdI*, and zeta-potential *ζ*. Parenthesized values were determined ca. 4 months after purchasing the AgNPs; all other values were determined directly after delivery. AF4 experiments were conducted within 2 months after AgNP delivery. Values shown in the table for 1:1 AgNP_LA mixtures were acquired with the best experimental conditions

**Sample**	***Batch characterization***					***AF4-UV/Vis-DLS system***			
	**AgNP (mg·L**^**−1**^**)**	***d***_***H***_ **(nm)**	***d***_***TEM***_ **(nm)** ^**a**^	***PdI***	***ζ*** **(mV)**	**Sample**	**AgNP (mg·L**^**−1**^**)**	**Carrier solution**	***d***_***H***_ **(nm)**
20 nm AgNP_LA	2.5	28.5 ± 1.0 (39.3 ± 1.7)	20.4 ± 3.0	0.24 ± 0.03 (0.32 ± 0.01)	– 26.7 ± 4.4	20 nm AgNP_LA^b^	50	Water pH 8	35
								0.05% v/v Mucasol	42
								1 mM NaCl	50
50 nm AgNP_LA	2.5	52.2 ± 0.6 (64.3 ± 2.3)	48.6 ± 4.5	0.12 ± 0.02 (0.20 ± 0.01)	– 20.1 ± 2.5	20 nm AgNP_LA in mixture 1:1^c^	50	Water pH 8	55
								0.05% v/v Mucasol	55
								1 mM NaCl	55
80 nm AgNP_LA	2.5	97.3 ± 0.8 (101.6 ± 1.1)	83.2 ± 10.4	0.07 ± 0.01 (0.09 ± 0.02)	– 32.0 ± 2.0	80 nm AgNP_LA in mixture 1:1^c^	50	Water pH 8	105
								0.05% v/v Mucasol	120
								1 mM NaCl	125

^a^Particle diameters (*d*_*TEM*_) determined with transmission electron microscopy were taken from the supplier’s specification sheet for the individual AgNP batches

^b^20 nm AgNP_LA *d*_*H*_ values for different carrier solutions (Fig. 3c)

^c^Different size fractions in 1:1 mixture of 20- and 80-nm AgNP_LA (Fig. 7)

The measured *d*_*H*_ for the three different AgNP batches were slightly higher than the diameters determined via transmission electron microscopy (*d*_*TEM*_) by the supplier. DLS measures hydrodynamic sizes, whereas TEM images are representative of hard-core sizes. The differences observed between the AgNP diameters measured using DLS and TEM are attributed to the LA coating thickness and polydispersity of the AgNP samples. The 80-nm AgNP_LA batch showed the narrowest and the 20-nm particles the widest distribution, with polydispersity indices (*PdI*) of 0.07 ± 0.01 and 0.24 ± 0.03, respectively. The determined negative zeta-potentials (*ζ*) (a measure for the formation of an electrochemical double layer and its strength) indicate that the coating with lipoic acid produced a negative surface charge by deprotonation of the carboxyl group. According to the DLVO theory [56, 57], this leads to an electrostatic stabilization of the particles. The long-term stability of the lipoic acid-coated AgNPs was investigated in terms of *d*_*H*_ and *PdI* after ca. 4 months of storage at 5 °C (parenthesized values in Table 2). For all three batches, *d*_*H*_ and *PdI* increased, especially for the 20- and 50-nm particles with a less negative zeta-potential and therefore weaker repulsion forces compared to the 80-nm particles. This observed increase in size and polydispersity of the AgNPs may be due to the highly concentrated stock dispersion (1 g/L), where the probability for a diffusion-controlled approach of the nanoparticles is much higher than that in dilute dispersions.

The different AgNP_LA batches featured absorbance spectra with absorbance maxima of *λ*_max_(*d* = 20 nm) = 399 nm, *λ*_max_(*d* = 50 nm) = 422 nm, and *λ*_max_(d = 80 nm) = 469 nm generated by surface plasmon resonance. The spectral position, peak height, and bandwidth of those characteristic absorption bands are determined by the particle composition, shape, and size as well as the dielectric characteristics of the surrounding environment [58]. The observable red shift of the absorption band with increasing particle diameter can be ascribed to surface plasmon resonances. Multipole resonances can be excited for particles with elevated diameters which leads to a reduction of the depolarization field, where electrons do not move in phase. This retardation effect results in a red shift of the absorption band [58]. The observable peak broadening and decrease of intensity with increased diameter can be ascribed to radiative losses, which contributes to the plasmon damping [58]. The recorded spectrum of a 1:1 mixture of 20- and 80-nm AgNPs (dashed line in Fig. 1) can be regarded as superposition of the two individual spectra. No shift of the peak position and bandwidth was observed when using different liquid media (ultrapure water, 0.05% v/v Mucasol and 1 mM NaCl; please be referred to Fig. S1). Thus, we did not find significant oxidation and/or aggregation of AgNP_LA in the studied media. Based on a previous study [55], we hypothesized that oxidation, in case it occurred, would take place after the aggregation and later sedimentation of the AgNPs.

The characteristic optical property of AgNPs of different sizes enables the determination of different AgNP size fractions using UV/Vis spectroscopy and is therefore a powerful detection tool for AF4 size separation approaches.

### AF4 experiments with individual AgNP batches

#### Quantity of injected particles

**Fig. 2:**
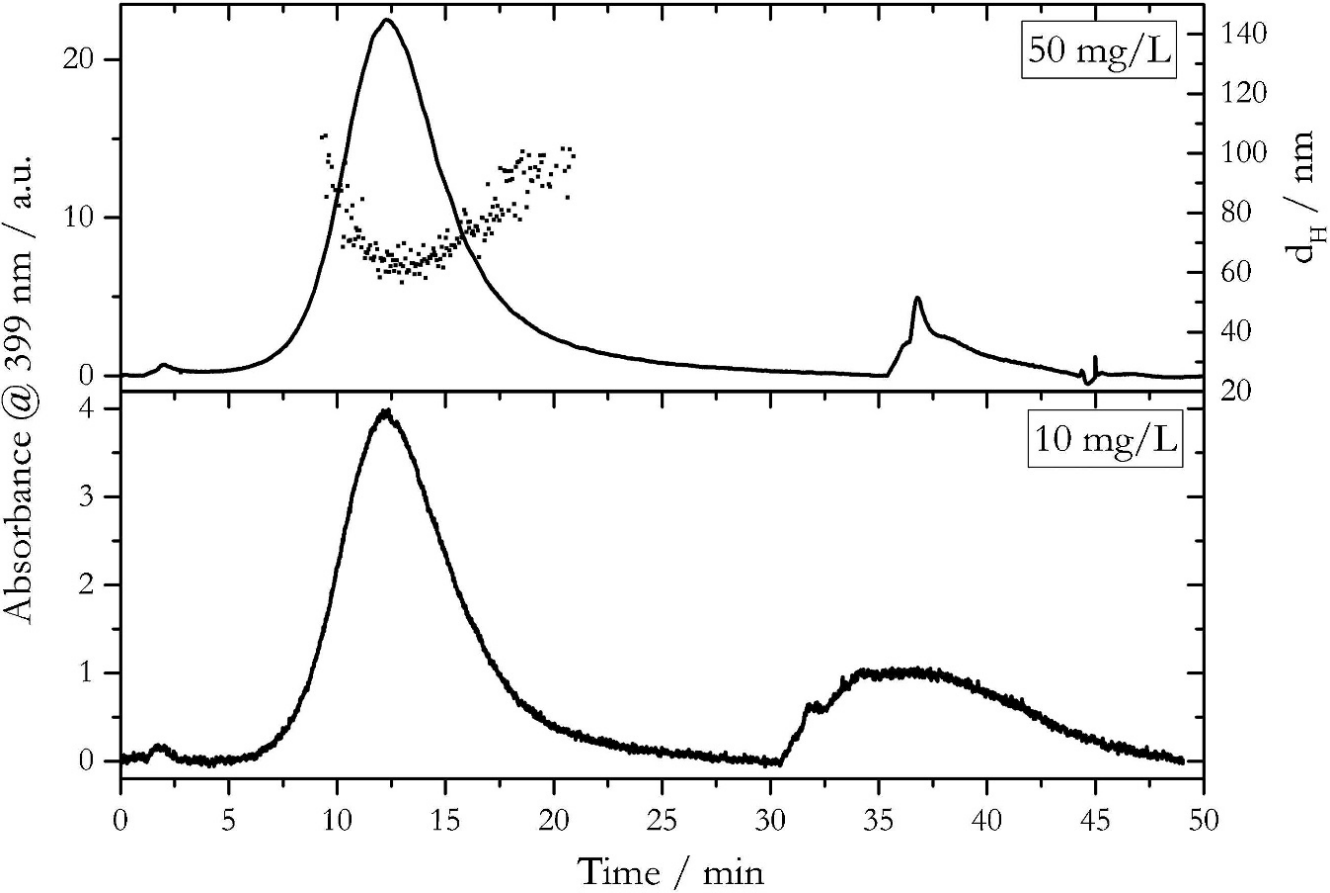
Fractograms of 20-nm AgNP_LA for 10 mg/L and 50 mg/L dispersions using a 20-μL injection loop, 1 mM NaCl as carrier solution, PES membrane, and a constant cross-flow of *V*_*x*_ = 0.5 mL/min. Dotted data points indicate hydrodynamic diameter (right y-axis)

In order to avoid a sample overload of the AF4 channel, it is recommended to inject 10^7^ to 10^10^ particles per run [59]. Higher particle numbers may result in a depression of retention time (*t*_*R*_) and an asymmetric shape (fronting or tailing) of the recorded peak [60–62]. With very low injected particle numbers, their detection can be negatively affected in terms of limit of detection and signal to noise ratio of the respective detection system. Fractograms for the injection of 10 and 50 mg/L dispersions of 20-nm AgNP_LA using the 20-μL injection loop, which is equivalent to the injection of 4.4·10^9^ and 2.2·10^10^ AgNPs, respectively, are shown in Fig. 2.

For both fractograms, a symmetric peak was obtained with a *t*_*R*_ of 12.1 min. The fractogram of the 10 mg/L dispersion showed a low signal to noise ratio compared to 50 mg/L, with five times lower peak intensity and area. Furthermore, the particle fraction could not be analyzed in terms of *d*_*H*_ via DLS as one can assume that the concentration was below the limit of detection, whereas DLS reading was obtained for the 50 mg/L dispersion. However, the recorded hydrodynamic diameter (with *d*_*H*_ = 60 nm at the minimum) is higher than the diameter determined in the batch experiments for the 20-nm AgNP_LA fraction (Table 2). This observation can be metrologically explained by a perturbation of the diffusion-controlled movement due to the applied flow in the online coupled DLS measurement cell [63]. Physico-chemical explanations for the observation of this bias will be given throughout the discussion below. Throughout our study, we observed a U-shaped distribution of the DLS data as a function of the elution time. This was also observed in a variety of other studies but the understanding of its appearance remains unclear. It can be hypothesized that the U-shaped distribution is related to low particle concentrations at the beginning and end of the elution peak, to the presence of larger aggregates in solutions or to a change of the shape of the particles during the separation and elution process [34]. In addition to the main AgNP peak, the AF4 fractograms feature void peaks (at *t*_*R*_ = 2 min in Fig. 2) due to unfocussed AgNPs, and release peaks (at *t*_*R*_ = 30 min for 10 mg/L and at *t*_*R*_ = 35 min for 50 mg/L dispersions in Fig. 2) due to retained AgNPs. The time shift of the release peak was caused by a 5 min earlier start of the flushing step for the 10 mg/L dispersion. The appearance and nature of the void and release peaks are further discussed throughout the following sections.

Based on the results shown in Fig. 2, we applied injections of 50 mg/L dispersions using a 20-μL loop, and of 10 mg/L dispersions using a 100-μL loop throughout the study presented herein. This was equivalent to a quantity of 2.2·10^10^ particles for 20-nm AgNP_LA, 1.7·10^9^ particles for 50-nm AgNP_LA, and 3.4·10^8^ particles for 80-nm AgNP_LA per injection.

#### Effect of carrier solution and applied cross-flow

**Fig. 3:**
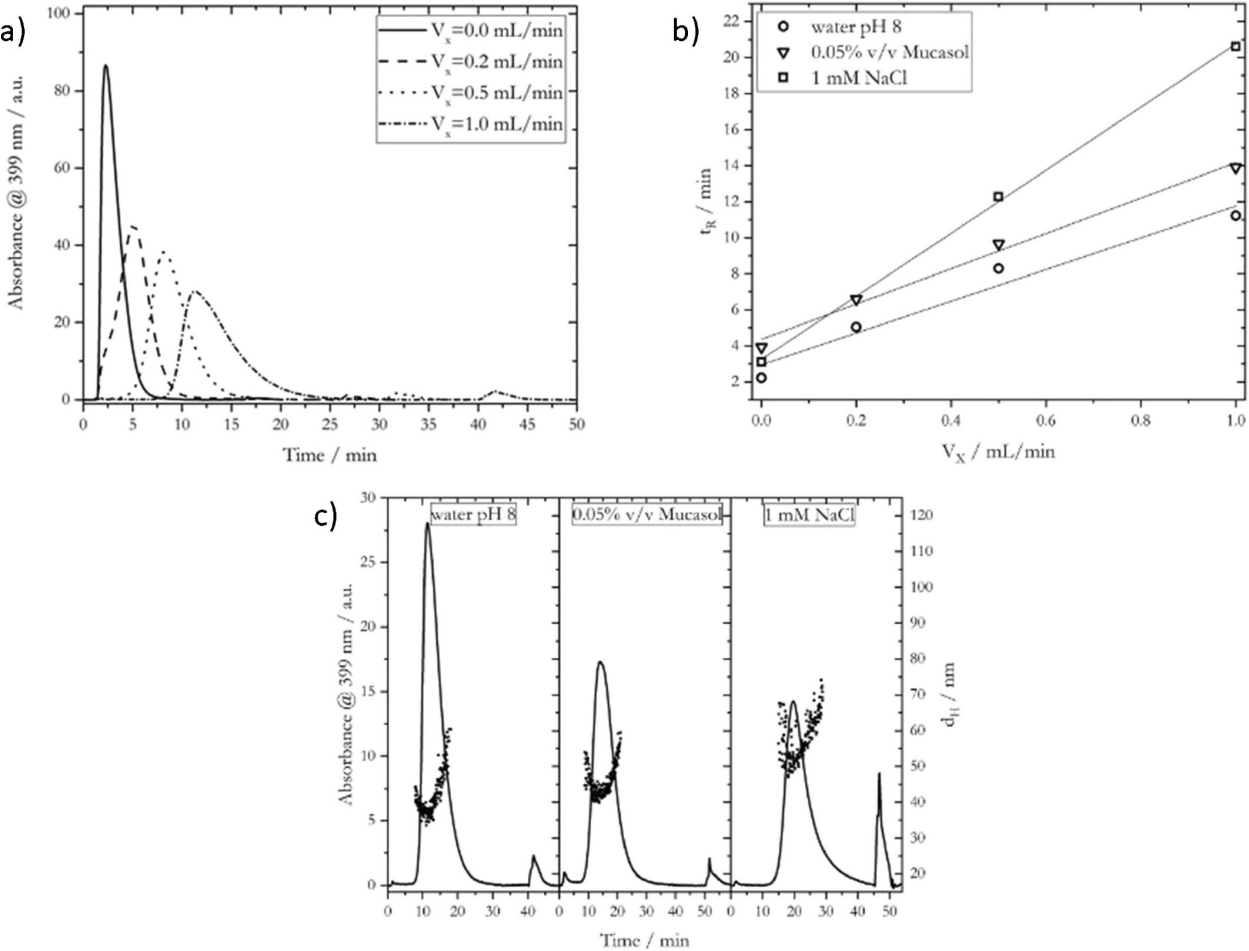
Effect of applied cross-flow and carrier solution on 20-nm AgNP_LA fractograms, retention time, and hydrodynamic diameter. **a** Fractograms obtained for different cross-flows and water pH 8 as carrier solution. **b** Retention time (*t*_*R*_) at different cross-flows and carrier solutions. **c** Fractograms and hydrodynamic diameter for different carrier solutions, *V*_*x*_ = 1.0 mL/min

The most characteristic feature of AF4 is the applied cross-flow (V_x_): a field directed perpendicular to the parabolic velocity channel flow profile which determines the nature and appearance of the peaks in AF4 fractograms. The effect of different cross-flows on the fractograms and *t*_*R*_ of the 20-nm AgNP_LA batch is shown in Fig. 3a and b. Fractograms for 20-nm AgNP_LA for 0.05% v/v Mucasol and 1 mM NaCl as carrier solution as well as data for 50- and 80-nm AgNP_LA batches are presented in Fig. S2 to Fig. S4.

A shift of the *t*_*R*_ as well as a broadening of the elution peaks was observed when increasing the applied cross-flow. The cross-flow affects the diffusion-driven transport of the injected particles perpendicular to parabolic velocity profile of the channel flow, and thus the vertical distance of the particles to the separation membrane. Particles of the same diameter are located closer to the separation membrane with increasing cross-flow, which means that they are affected by lower channel flow rate and broader velocity distribution, resulting in slower elution (longer *t*_*R*_) and broadening of the elution peak. The relationship between *t*_*R*_ and applied cross-flow as a function of particle diameter can be assumed to be linear (Fig. 3b) according to the following first-order approximation [60]:

1 $$ {t}_R=\frac{\pi \eta {w}^2{t}^0{d}_H}{2{k}_BT{V}^0}\cdotp {V}_x, $$

with the solvent viscosity *η*, the channel height *ω*, the time required for the carrier solution to pass through the channel (void time) *t*^*0*^, the Boltzmann constant *k*_*B*_, the temperature *T*, and the geometric volume of the channel *V*^*0*^. Based on this equation, particles of different diameter feature a characteristic *t*_*R*_, which enables a size separation via AF4 (see section “AF4 experiments with AgNP mixtures”).

Another important parameter, which affects the performance of AF4 systems, is the selected carrier solution. It can be easily modified in terms of composition, ionic strength, and pH value in order to ensure good quality of acquired fractograms and size separation of polydisperse nanoparticle samples. For AF4 experiments, it is recommended to use carrier solutions in a near-neutral pH range to ensure safe system operating conditions [59]. Moreover, the physico-chemical properties of the carrier solution should nearly mimic the properties of the injected nanoparticle sample in order to avoid perturbations such as aggregation, changes in particle and membrane charge or double-layer thickness and particle-membrane interactions [26]. Because of our future goal to characterize seawater samples, a pH of ~8 was chosen for ultrapure water and 1 mM NaCl as carrier solutions. Additionally, a carrier solution containing 0.05% v/v of the alkaline surfactant Mucasol was tested. Surfactants were applied in several studies in order to control the interactions between particles and membrane [26, 59]. In general, the less the interactions particle-membrane and particle-particle, the shorter *t*_*R*_ [64–66].

The 20-nm AgNP_LA batch showed the highest *t*_*R*_ with 1 mM NaCl as carrier solution, and the lowest with water pH 8 (Fig. 3b and c). This observation was consistent throughout the different AgNP_LA batches (see Fig. S5). As the ionic strength is increased, the electrochemical double layer is more compressed of both the negatively charged PES membrane (*ζ* ~ −10 mV was assumed for the PES membrane at pH ~ 8 [67]) and the negatively charged AgNP_LA batches. This compression resulted in a shorter Debye length and therefore a shorter vertical distance between particles and membrane [59]. As the particles approached closer to the membrane surface due to less repulsive forces, they were affected by a slower channel flow rate and broader velocity distribution, which caused longer *t*_*R*_ and peak broadening. When using 0.05% v/v Mucasol as carrier solution, slightly longer *t*_*R*_ compared to water pH 8 but noticeable shorter times compared to 1 mM NaCl were observed (Fig. 3b and c). Any statement about the ionic strength of the Mucasol-containing carrier solution is difficult due to the unknown concentrations of the containing ionic species in solution. However, the pH of the 0.05% v/v Mucasol solution (pH = 10.5) is 2.5 pH units higher than the values for the other used carrier solutions. This pH increase could have caused a shift in the zeta-potential of the PES membrane of around 5 to 10 mV to more negative values, compared to using solutions at pH ~ 8 [67]. The same effect on the zeta-potential was observed for negatively charged AgNPs when increasing the pH value of the surrounding solution [68]. This higher charge of both the PES membrane and the AgNPs when using 0.05% v/v Mucasol at pH = 10.5 led to stronger repulsive forces and therefore shorter *t*_*R*_ compared to 1 mM NaCl. Besides the shift in *t*_*R*_ due to the compression of the electrochemical double layer, the *d*_*H*_ of the particles was also increased when using carrier solutions with a higher ionic strength than water pH 8, as shown in Fig. 3c. Increases of about 7 nm and 15 nm of *d*_*H*_ were observed for 0.05% v/v Mucasol and 1 mM NaCl, respectively. According to the DLVO theory, increased ionic strength destabilizes electrostatically stabilized systems, thus favoring the particle aggregation and causing an increase of *d*_*H*_ and *t*_*R*_. The more negative charge of the particles when using 0.05% v/v Mucasol compared to 1 mM NaCl, due to the different pH values of the solutions, caused stronger repulsive forces between the AgNPs and thus an attenuation of the aggregation process.

#### Effect of focus time

**Fig. 4:**
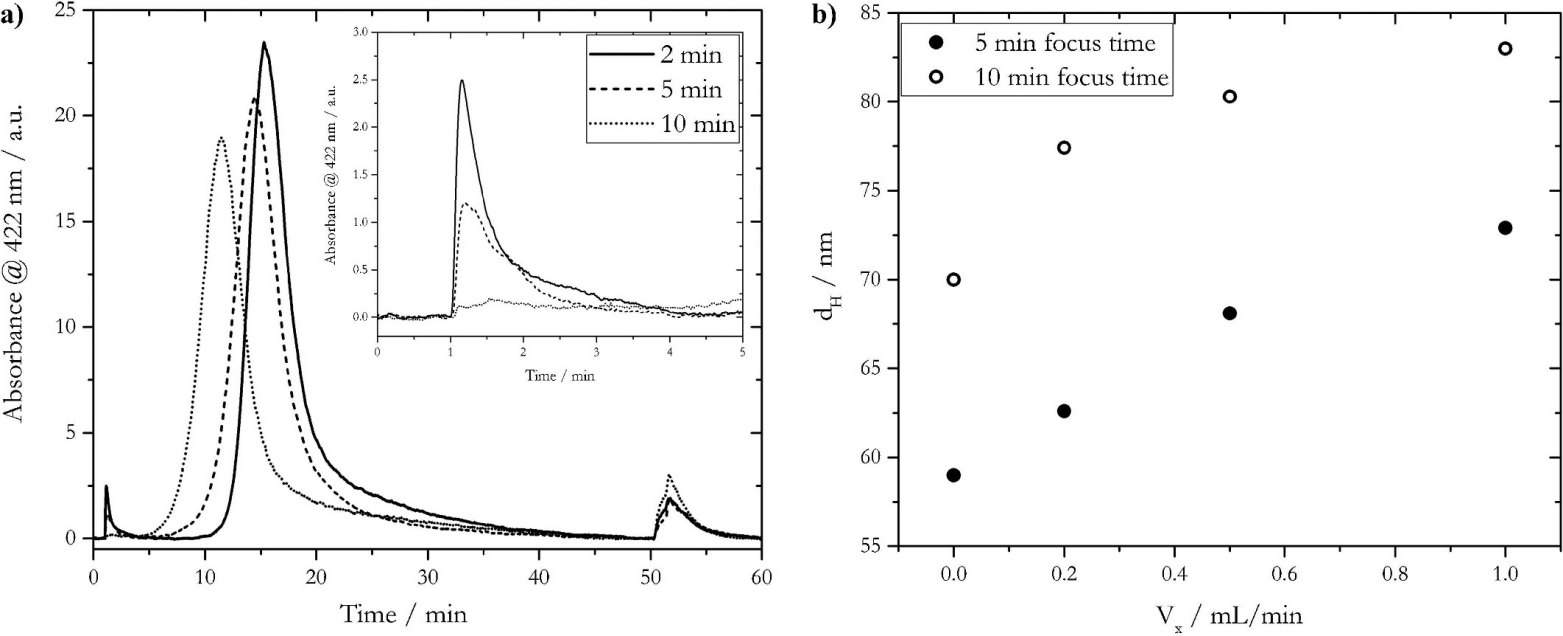
Effect of different focus times on the fractograms and *d*_*H*_ of 50-nm AgNP_LA with water pH 8 as carrier solution. **a** Fractograms acquired with *V*_*x*_ = 0.5 mL/min; inset shows the void peak. **b** Hydrodynamic diameters determined with online coupled DLS at *t*_*R*_ of absorbance maximum for different applied cross-flows and focus times

The focusing step in AF4 experiments (Table S1, step 4) ensures that injected particles will be focused sufficiently in a horizontal zone which is as small as possible [69]. Furthermore, and most important, a vertical relaxation of the injected particles takes place as a function of their individual diffusion coefficients and applied cross-flow [70]. Thus, the duration of the focusing step and the applied cross-flow affects the nature and quality of the obtained fractogram [71] as shown in Fig. 4.

The signal of the void peak decreased with longer focus times (inset of Fig. 4a). For example, the signal of the void peak for a focus time of 5 min was only half the intensity of the peak acquired with a focus time of 2 min in step 4, while the appearance of a void peak was almost negligible for 10 min of focusing time. This observation can be ascribed to an insufficient focus step for shorter focus times, leading to the pre-elution of unfocused nanoparticles. Additionally, a delayed elution of the 50-nm AgNP_LA batch with shorter focus times was observed, e.g., the elution peak for 10-min focus time had its maximum at 11.5 min, whereas the maximum for 2-min focus time was at 15.3 min. During injection, particles are accumulated near the membrane on the bottom of the channel. Once the injection flow is switched off, relaxation processes affect the particles producing a diffusion-controlled transport of the particles towards the center of the channel in accordance with their respective diffusion coefficients. If this time for relaxation (focus time) is chosen too short, insufficient relaxed particles are affected by slower speed of the carrier solution during the elution step, compared to well-relaxed particle spots. This occurs due to a closer distance of the particles to the membrane, and the parabolic velocity profile. The focus time not only affects the position of the elution peak, but also its signal intensity. It is assumed that particles are more favorable for the attachment on the membrane in the focusing zone as a consequence of longer applied high field strengths [60]. This is in accordance with a more intense release peak for a focus time of 10 min compared to smaller release peaks when shorter focus times were applied (at *t*_*R*_ > 50 min in Fig. 4a). Release peaks are generated due to reversibly attached particles to the membrane, which are eluted in the flushing step when no cross-flow is applied.

The duration of the focus time also affected the *d*_*H*_ of the detected fractions, as shown in Fig. 4b. When there was no cross-flow and a 5-min focus step was applied, particles with *d*_*H*_ = 59 nm were detected via online DLS. In contrast, a 10-min focusing step led to the detection of a AgNP size fraction with *d*_*H*_ = 70 nm. This observed increase of *d*_*H*_ was caused by a higher probability of particle aggregation due to high focus field strengths, coming along with higher particle concentration in a very small zone and therefore closer particle-particle distance [60]. The same effect was observed as a consequence of an increased applied cross-flow (Fig. 4b). The *d*_*H*_ was ca. 15 nm higher with *V*_*x*_ = 1.0 mL/min, compared to no applied cross-flow for both 5-min and 10-min focus times.

#### Recovery

**Fig. 5:**
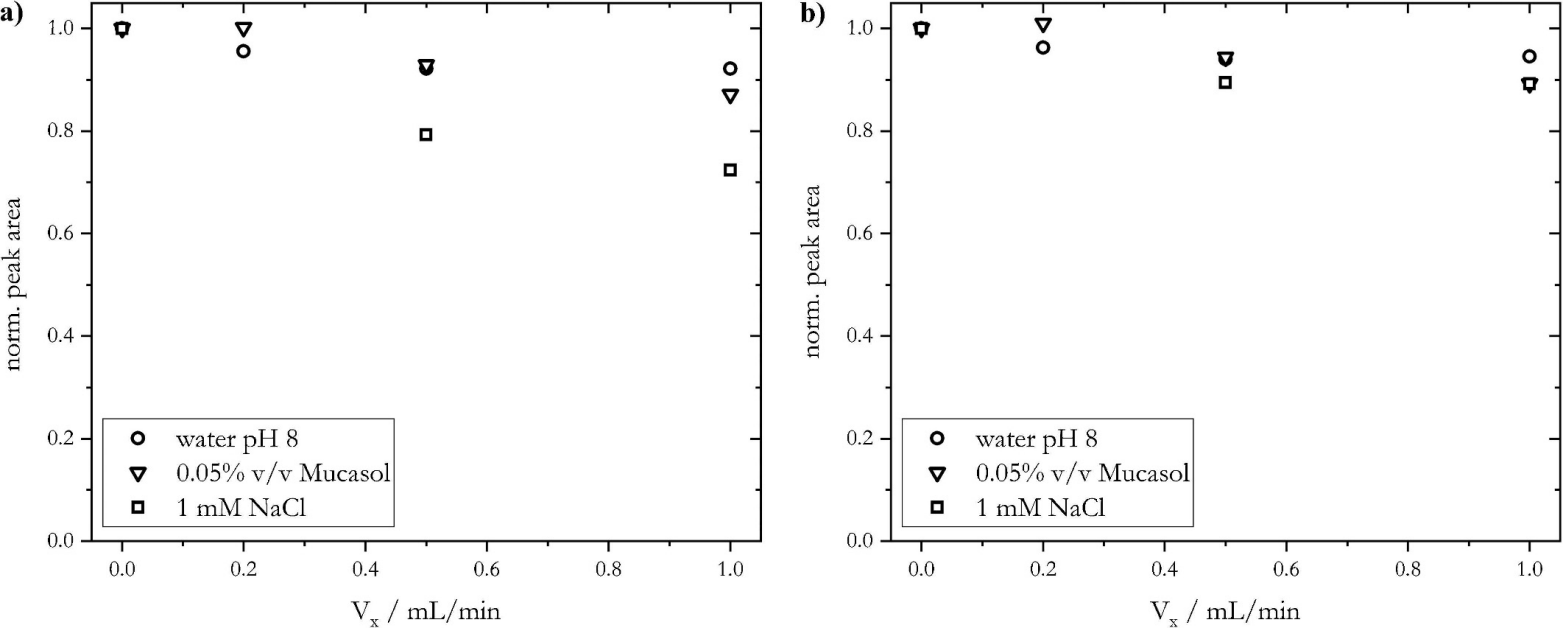
Recovery, expressed as normalized peak area, of 20-nm AgNP_LA for different carrier solutions: ultrapure water at pH 8 (circles), 0.05% Mucasol (triangles), and 1 mM NaCl (squares) as a function of the applied cross-flow

The recovery of the sample is a suitable parameter for the evaluation of the loss of particles as a consequence of their accumulation on the separation membrane due to applied cross-flows in AF4 experiments. Figure 5 shows the recovery for 20-nm AgNP_LA with different carrier solutions as a function of the applied cross-flow. Here, the recovery is expressed as a normalized peak area, with a normalized peak area of 1.0 being equivalent to a 100% recovery. Normalized peak areas were obtained from the respective fractograms by dividing the area of the elution peak (Fig. 5a) or the sum of the areas of elution and release peak (Fig. 5b) by the area obtained for *V*_*x*_ = 0.0 mL/min. Thus, it was assumed that 100% recovery was achieved when no cross-flow was applied.

Increased cross-flows affected the recovery of the 20-nm AgNP_LA sample negatively: ca. 10% decrease in recovery for cross-flows of 0.5 and 1.0 mL/min, when water pH 8 and 0.05% v/v Mucasol were used as carrier solutions. Previous studies demonstrated the effect of high cross-flows on the increment of the membrane fouling, and therefore the decrease of the sample recovery [72]. This can be explained due to more favorable inter-particle associations, and both reversible and irreversible particle adhesion to the membrane because of high field strengths [73, 74]. This effect was even more predominant when using 1 mM NaCl as carrier solution with recoveries of 80% and 73% for *V*_*x*_ = 0.5 and 1.0 mL/min (Fig. 5a), respectively, as a consequence of an elevated ionic strength affecting particle-particle and particle-membrane interactions. Yet a considerable AgNP fraction was reversibly attached to the membrane, resulting in the presence of a dominant release peak, and thus a shift of recovery to values up to 90% when taking into account the sum of the area of the elution and release peaks (Fig. 5b). The reversibly attached particles on the membrane, either in the focusing zone or in the separation zone, were released when turning off the cross-flow in the flushing step (step 6, Table S1). This re-entrainment produced a release peak in the respective fractograms (Fig. 3a and Fig. S2). The area of this peak increased with increasing cross-flows. It was further observed that the sum of both areas cannot be correlated to a recovery of 100% with applied cross-flow for all carrier solutions (Fig. 5b). Thus, the presence of irreversibly attached particles or aggregation processes can be assumed, most prominently in the focusing zone, where high field strengths were applied [60]. In this zone, a change of color of the membrane was observed after several runs by a visual inspection, supporting the argument of irreversible accumulation of AgNPs.

### AF4 experiments with AgNP mixtures

#### Quality of separation

**Fig. 6:**
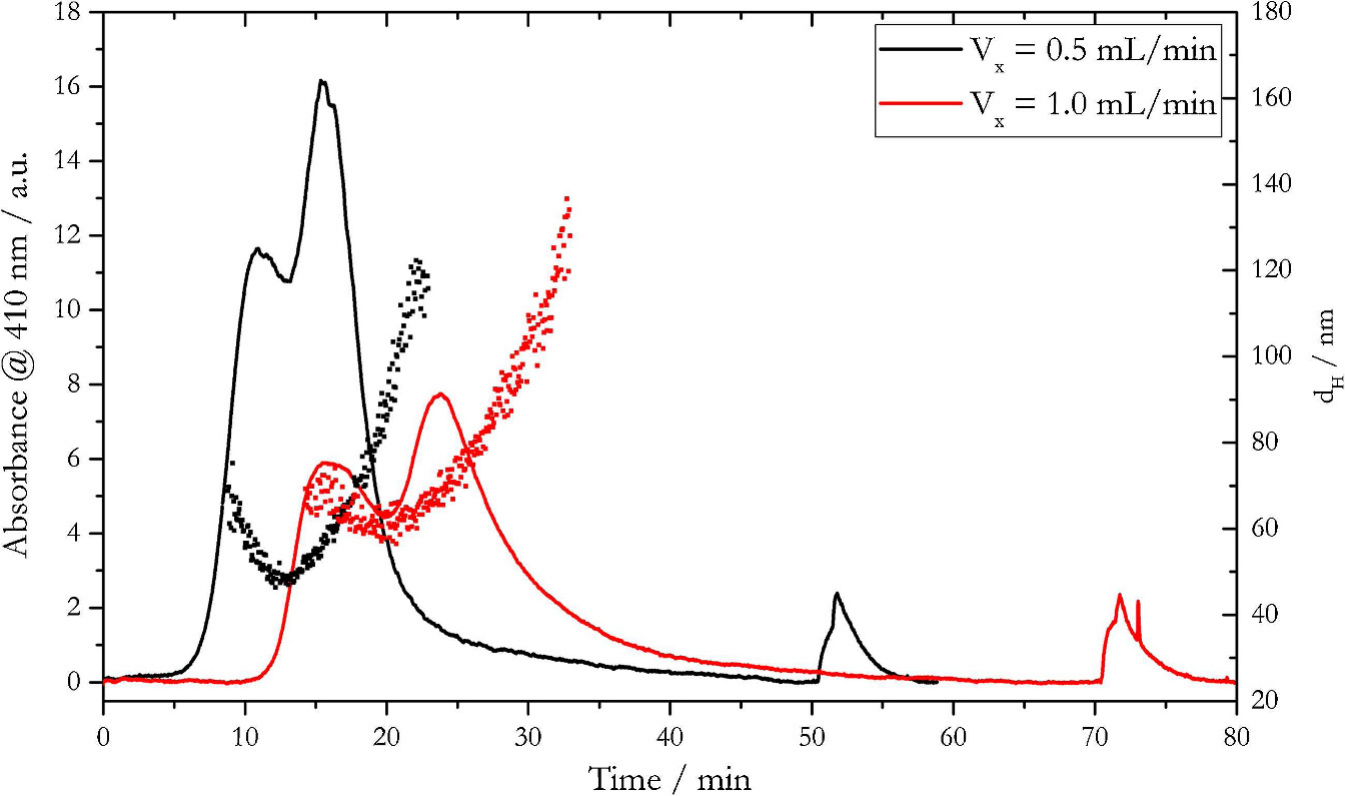
Fractograms and online determined hydrodynamic diameter of a 1:1 mixture of 20- and 50-nm AgNP_LA for different cross-flows and water pH 8 as carrier solution

Based on Eq. (1) and the results presented in section 3.2, fractograms of a mixture of particles of two different sizes should feature two distinct, and most preferable baseline separated elution peaks, each referring to one particle size fraction. Figure 6 shows fractograms for a 1:1 mixture of 20- and 50-nm AgNP_LA particles at two different cross-flows.

Although two maxima were obtained for each fractogram, corresponding to 20- and 50-nm particles, the two elution peaks overlapped, which indicates an insufficient separation of the size fractions, for both *V*_*x*_ = 0.5 mL/min and *V*_*x*_ = 1.0 mL/min. The height of the absorption signals decreased by ca. 6 units for a cross-flow of 1.0 mL/min compared to 0.5 mL/min. This was in accordance with previous observations that a higher cross-flow generates a higher loss of AgNPs due to the attachment on the membrane and more favorable particle-particle interactions. The existence of aggregated species was also indicated by the obtained DLS signal. The *d*_*H*_ of the mixture of particles increased by 12 nm at the minimum, showing a broader distribution for *V*_*x*_ = 1.0 mL/min, when compared to *V*_*x*_ = 0.5 mL/min. Considering *d*_*H*_, no distinct separate signals for the 20- and the 50-nm AgNP_LA size fraction were obtained. Thus, it can be assumed that the *d*_*H*_s as well as the individual diffusion coefficients were too similar, which precluded an efficient separation. Presumably, higher applied cross-flows would have led to a better separation, but at the expense of much longer elution times and much greater particle losses due to aggregation and membrane attachment.

**Fig. 7:**
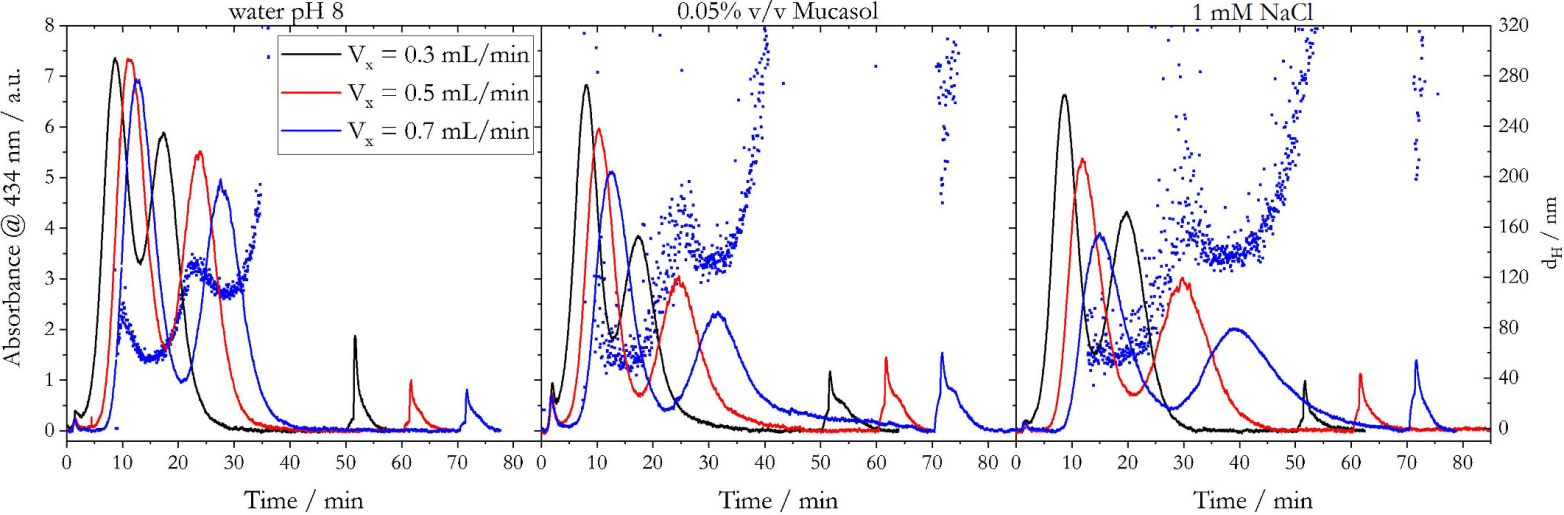
Fractograms of a 1:1 mixture of 20- and 80-nm AgNP_LA acquired with different cross-flows and carrier solutions. Hydrodynamic diameter was determined for *V*_*x*_ = 0.7 mL/min

A better size separation of AgNPs was achieved when using a 1:1 mixture of 20- and 80-nm AgNP_LA (Fig. 7).

The maxima of the elution peaks for the individually applied cross-flows and carrier solutions were in good agreement with the *t*_*R*_ obtained for the single AgNP_LA batch experiments (e.g., Fig. S5). Longest *t*_*R*_ was determined with 1 mM NaCl and earliest elution was observed with water pH 8 as carrier solution due to the different ionic strengths of the solutions, affecting particle-particle and particle-membrane interactions. Best separation of 20-nm and 80-nm AgNP_LA was achieved with the carrier solutions 0.05% v/v Mucasol and 1 mM NaCl and applied cross-flows of ≥0.5 mL/min. Under those conditions, the two peaks were separated considerably closer to the baseline than for *V*_*x*_ = 0.3 mL/min and water pH 8 as carrier solution. The absorbance values at the minimum between the two separated elution peaks were 0.6 and 0.4 a.u. for the applied cross-flows of 0.5 mL/min and 0.7 mL/min, respectively, for both 0.05% v/v Mucasol and 1 mM NaCl. However, the slightly better separation for *V*_*x*_ = 0.7 mL/min compared to 0.5 mL/min came with much longer durations (10 to 15 min) of the separation experiments. The fractograms obtained with 0.05% v/v Mucasol as carrier solution feature a much more intense void peak (at *t*_*R*_ = 2 min) than fractograms for water pH 8 and 1 mM NaCl, as also observed in the single batch experiments (e.g., Fig. 3c and Fig. S2 to S4). This feature may be explained by the formation of micelles. The applied high field strengths during the focusing step could have induced a pre-concentration of the surfactant molecules, which exceeded the critical micelle formation concentration. It was found in earlier studies that the formation of micelles can generate an undesired elution, which interfere with the detection of nanoparticles [59].

Higher tendency for particle aggregation can be assumed for 0.05% v/v Mucasol and 1 mM NaCl compared to water pH 8, which agrees with the lower detected intensity of the peaks for these carrier solutions and shift to longer elution times (Fig. 7). Those aggregates were also detectable with the hyphenated DLS detector (*d*_*H*_ in Fig. 7). For all carrier solutions, two distinct signals were obtained for *d*_*H*_, correlating to the separated 20-nm and 80-nm AgNP_LA fractions. However, compared to water pH 8, *d*_*H*_ of the 80-nm fraction was increased by ca. 15 nm and 20 nm when using 0.05% v/v Mucasol and 1 mM NaCl, respectively, due to favorable aggregation processes as a consequence of the increased ionic strengths of the carrier solutions. The aggregation was also linked to the detection of a broader size distribution for both 20-nm and 80-nm AgNP_LA fractions when using 0.05% v/v Mucasol and 1 mM NaCl as carrier solutions.

#### Separation of AgNPs in marine coastal waters as a case study

**Fig. 8:**
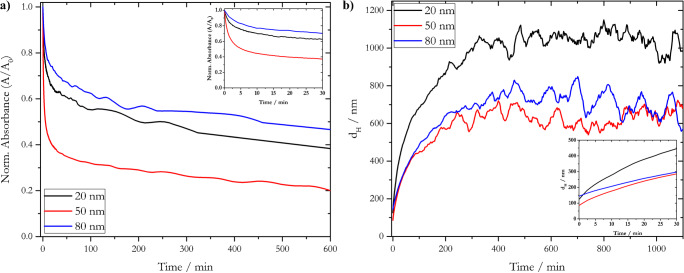
Temporal trend of **a** the normalized absorbance of the SPR band (calculated by dividing the absorbance (A) at the given time after spiking AgNP_LA into fjord water by the absorbance at *t* = 0 min (A0)) measured with UV/Vis spectrophotometry and **b** hydrodynamic diameter measured with DLS

As AF4 is a sensitive technique to determine distinct signals for different nanoparticle size fractions, we assumed that this technique could be used to investigate the fate of AgNPs, in terms of their aggregation behavior, in natural waters. First, we characterized the behavior of AgNP_LA in the fjord water sample in batch using the DLS and UV/Vis techniques (Fig. 8). The size evolution over time was measured to check for possible NP aggregation during the AF4 experiments.

The normalized absorbance of the SPR band for 50-nm AgNP_LA featured the steepest decrease over time (red line, Fig. 8a), indicating a low stability compared to 20- and 80-nm AgNP_LA. Slowest decrease was observed for 80-nm AgNP_LA (for absorbance spectra, see Fig. S6a, b, c). This observation is in accordance with the determined zeta-potentials (Table 2), where more negative values indicate enhanced stability. Normalized absorbance showed little change after ca. 10 min for all three AgNP_LA fractions (inset Fig. 8a). The original *d*_*H*_ values (Table 2) were increased over time up to values around 600 and 1000 nm for *d*_*H*_ = 50/80 and 20 nm, respectively (for temporal trend of *PdI*, see Fig. S6d). The observed behavior is similar to that found with other electrostatically stabilized AgNPs in marine environments [20], and is attributed to the weak electrostatic stabilization provided by specific coatings such as the lactic acid, and potential interactions with the organic matter present in the sample. The observed transformations of the pristine AgNP_LA involved aggregation and possibly oxidation processes, though a detailed study of these phenomena is out of the scope of this manuscript. Using an analytical approach that combined AF4, ICP-MS, and UV/Vis, D.C. António et al. [75] showed that it is possible to assess in detail the agglomeration process of electrostatically stabilized AgNPs in artificial seawater.

**Fig. 9:**
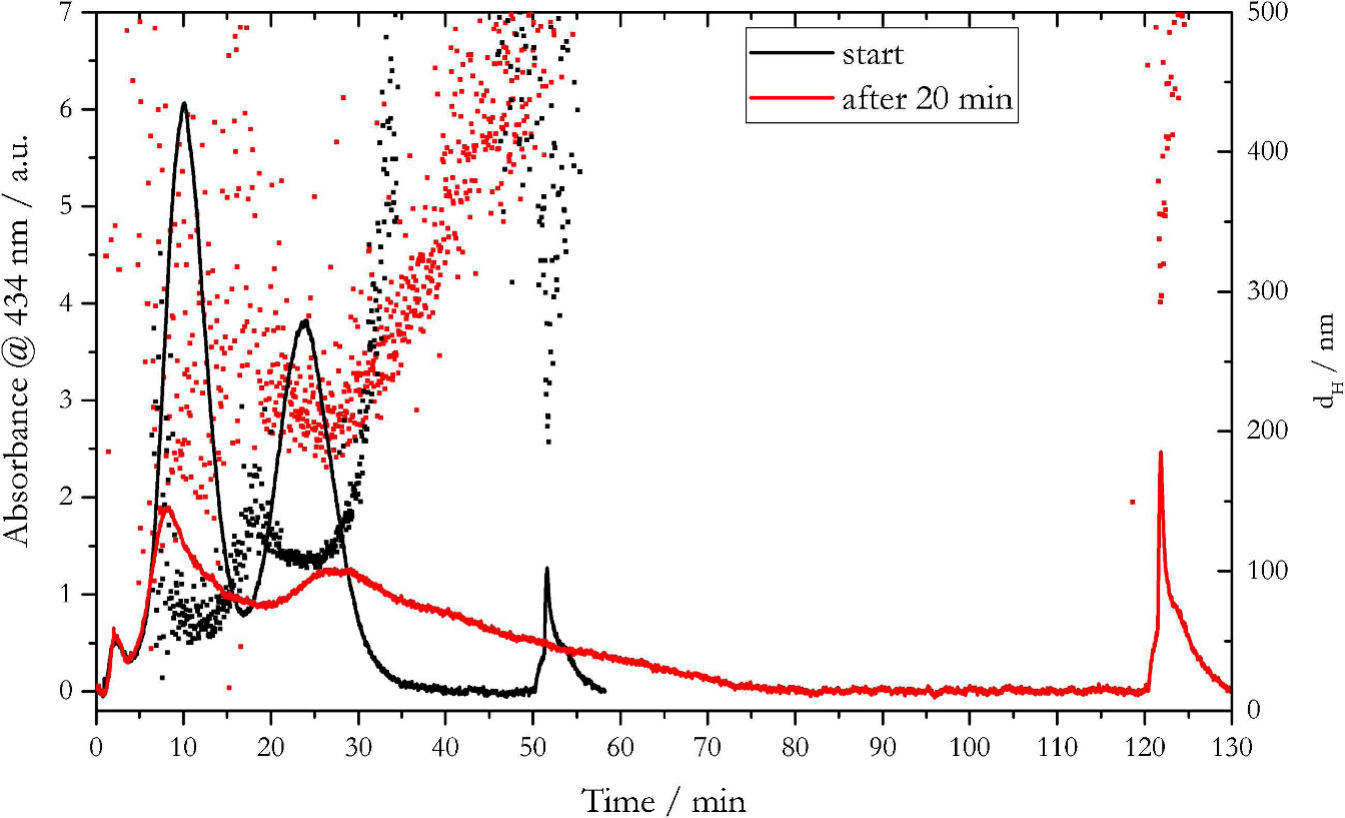
Fractograms and hydrodynamic diameter of a 1:1 mixture of 20-nm and 80-nm AgNP_LA acquired with *V*_*x*_ = 0.5 mL/min and 0.05% v/v Mucasol as carrier solution. Black color refers to a mixture prepared in ultrapure water; red color refers to the preparation in fjord water and injection into the AF4 system 20 min after preparation

As a case study of the AF4 technique optimization, a 1:1 mixture of 20- and 80-nm AgNP_LA was spiked into ultrapure water and marine water collected from Kiel Fjord. The dispersion prepared in ultrapure water was directly injected into the AF4 system once prepared (black signal in Fig. 9). In order to track any changes in elution behavior/particle size with the change of the dispersion matrix, the AgNPs dispersed in marine water (red signal in Fig. 9) were injected into the AF4 system 20 min after the preparation, when small aggregates were already formed in the solution (Fig. 8). Based on the AF4 optimization experiments, 0.05% v/v Mucasol as carrier solution and *V*_*x*_ = 0.5 mL/min were chosen for this case study as a compromise between separation quality and duration of the separation run.

A decrease in the elution peak height of ca. 66% was observed for both fractions, from 6.0 to 2.0 a.u. for 20-nm AgNP_LA and from 3.8 to 1.3 a.u. for 80-nm AgNP_LA. This decrease was accompanied by a broadening of the 80-nm AgNP_LA elution peak and a shift to longer elution times, from 24 min with mixtures prepared in ultrapure water to about 28 min with AgNPs spiked in fjord water. In contrast, the *t*_*R*_ for 20-nm AgNP_LA in fjord water decreased slightly (8.2 compared to 10.2 min for AgNP_LA dispersed in ultrapure water). In addition, an increase in the release peak was observed. This fact is explained by the formation of larger size particle fractions in high ionic strength matrices like the fjord water. Under cross-flow conditions, these aggregated fractions are completely retained in the separation channel and are released at the end of the elution process. DLS data showed poorly separated signals when AgNPs were spiked into natural fjord water, with an almost extinction of the 20-nm AgNP_LA fraction, and a shift of the 80-nm AgNP_LA size fraction signal from 100 to 200 nm, accompanied with much broader size distribution. In this case, a potential contribution from the dissolved organic matter in the fjord water should be also considered, thus limiting the applicability of this approach to similar matrices than those used in this study.

## Conclusions

This study showed that AF4 coupled with a multiparameter detection system can be a powerful tool to investigate the behavior and fate of nanoparticles in natural waters. A thorough characterization and optimization of the system is required in order to determine the best possible conditions for a careful and reliable examination of AgNPs. It was shown that parameters such as quantity of injected particles, applied cross-flow, focusing time, and carrier solutions affect the nature of the acquired fractograms and thus the quality of the size separation of AgNPs. Furthermore, parameters for size separation have to be chosen in a manner that integrity of the AgNPs within the separation system will not be jeopardized in terms of particle-particle and particle-membrane interactions. However, a compromise has to be made between high sample throughput and separation quality, as best separation was achieved under high cross-flow conditions which came along with longer experiment times. Thus, the behavior and fate of AgNPs in natural waters, in terms of their aggregation kinetics, can be recorded using hyphenated AF4-UV/Vis-DLS, but at the cost of temporal resolution. The AF4 technique can be used for the direct injection of marine water samples to characterize stabilized silver nanoparticle species at concentrations exceeding those currently found in natural waters.

## Supplementary information

ESM 1 (DOCX 1224 kb)

## Data Availability

The datasets generated during and/or analyzed during the current study are available from the corresponding authors on reasonable request.
