# Supplementary material for: Optimization of hyphenated asymmetric flow field-flow fractionation for the analysis of silver nanoparticles in aqueous solutions
Source: Anal Bioanal Chem. 2021 Sep 19;413(27):6889–904. doi: 10.1007/s00216-021-03647-3 (PMC8449749; doi:10.1007/s00216-021-03647-3)
Supplement: Supplementary file 1 — (DOCX 1224 kb) [file 216_2021_3647_MOESM1_ESM.docx]

**Supplementary Information: Optimization of hyphenated asymmetric flow field-flow fractionation for the analysis of silver nanoparticles in aqueous solutions**

**Felix Geißler^1^, María Martínez-Cabanas^1^*, Pablo Lodeiro^1,2^ and Eric P. Achterberg^1^**

^1^ Chemical Oceanography, Marine Biogeochemistry, GEOMAR Helmholtz Centre for Ocean Research Kiel, Kiel, Germany

^2^ Department of Chemistry, University of Lleida – AGROTECNIO-CERCA Center, Rovira Roure 191, 25198, Lleida, Spain

* Correspondence: M.M.C.: [mmartinez@geomar.de](mailto:mmartinez@geomar.de)

**1. Example of an AF4 run program**

Table S 1. Cross-flow profile for the separation of AgNPs using AF4.

| **No.** | **Step** | **Duration [min]** | ***V_x_* [mL/min]** | **Remarks** |
| --- | --- | --- | --- | --- |
| 1 | Elution | 1 | 0.7^1^ | Pre-conditioning |
| 2 | Focus | 1 | 2.0 |  |
| 3 | Focus + Inject | 1 or 2 | 2.0 | Handling of AgNPs |
| 4 | Focus | 10^1^ | 2.0 |  |
| 5 | Elution | 20^1^ | 0.7^1^ |  |
| 6 | Elution + Inject | 1 | 0.0 | Flushing |

^1^ Throughout the study presented herein, these parameters were subject of modification in order to determine the optimum conditions for the separation of AgNPs.

The purpose of the pre-conditioning steps 1 and 2 was to provide a baseline and to stabilize the pressure inside the system. In step 3 to 5 the AgNPs were injected onto the separation membrane, focused and separated according to their individual diffusion coefficients. For the injection of the sample (step 3) a duration of one minute for the 20 µL and two minutes for the 100 µL injection loop was chosen in order to ensure a complete release of the whole sample into to separation channel. The acquisition of the fractograms started with the elution step 5. The system was flushed without any cross-flow in step 6 to remove any remaining AgNPs from the separation system, observable as a ‘release peak’ in the fractograms.

**2. UV/Vis spectra**

| 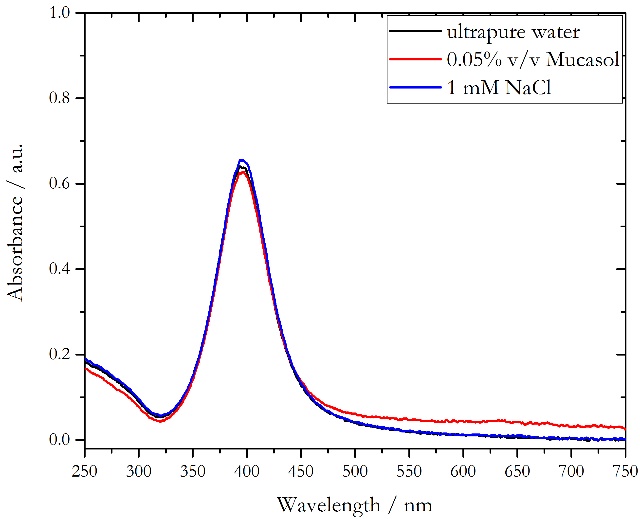 | 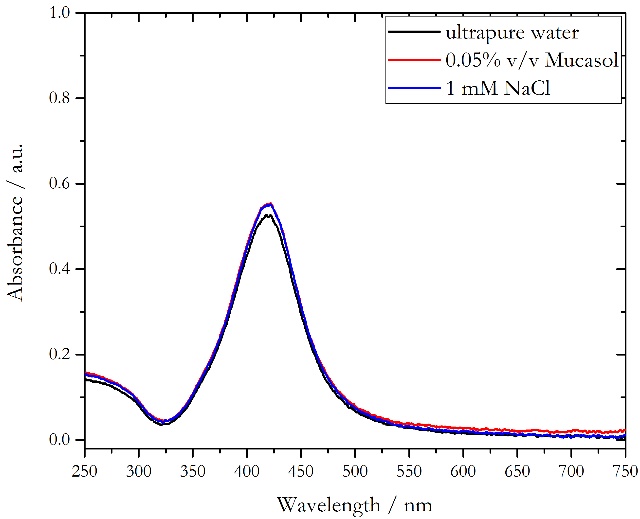 |
| --- | --- |
| (**a**) 20 nm AgNP_LA | (**b**) 50 nm AgNP_LA |
| 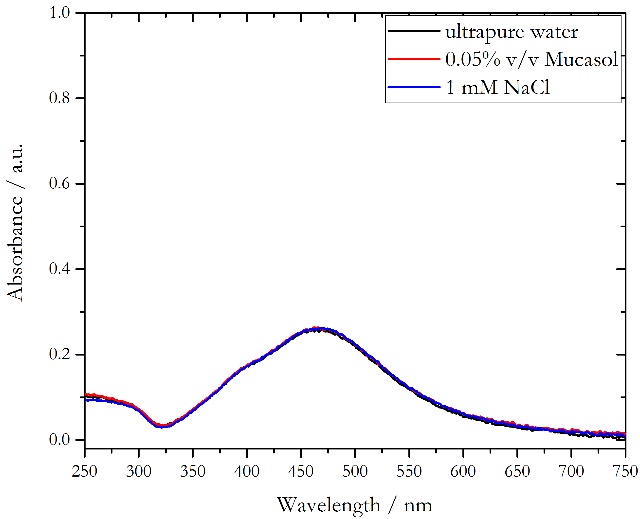 | 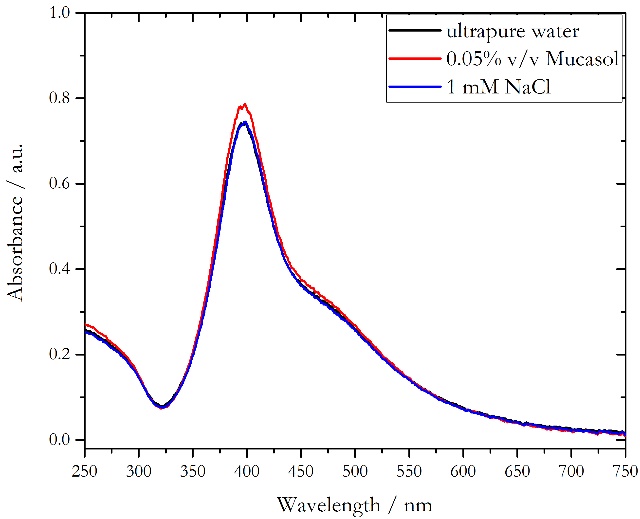 |
| (**c**) 80 nm AgNP_LA | (**d**) mixture of 20 nm and 80 nm AgNP_LA |

Fig. S1 UV/Vis spectra of AgNPs of different diameters coated with lipoic acid in three different liquid media.

**3. Fractograms of 20 nm, 50 nm and 80 nm AgNP_LA**

| 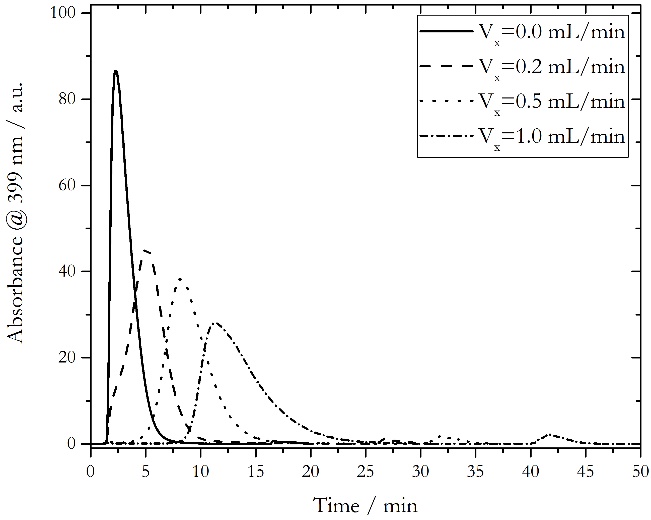 | 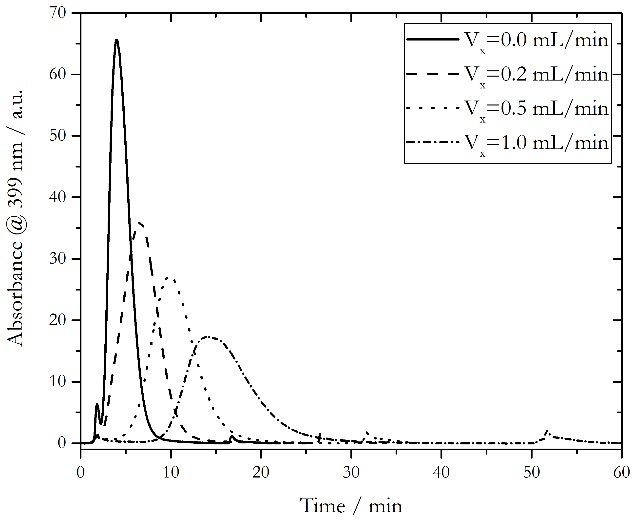 |
| --- | --- |
| (**a**) water pH 8 | (**b**) 0.05% v/v Mucasol |
| 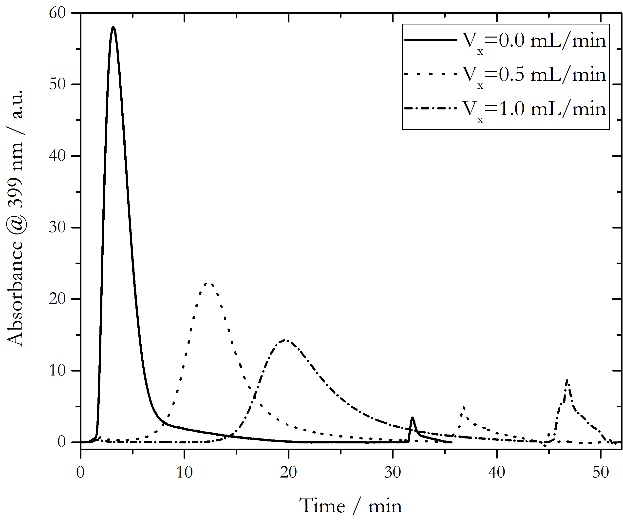 | |
| (**c**) 1 mM NaCl | |

Fig. S2 Fractograms of 20 nm AgNP_LA for different applied cross-flows and different carrier solutions.

| 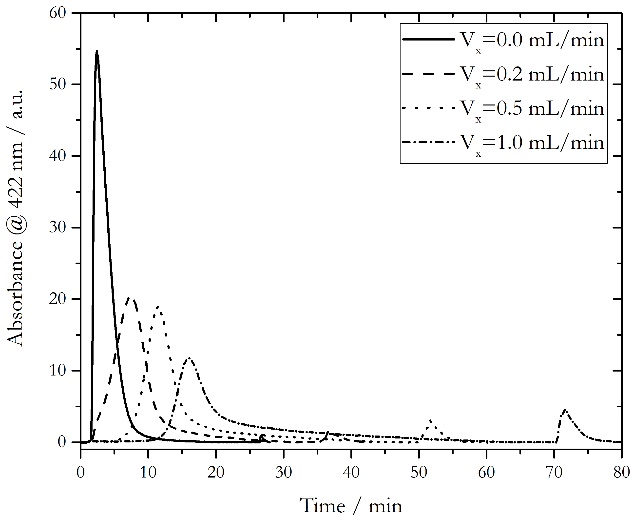 | 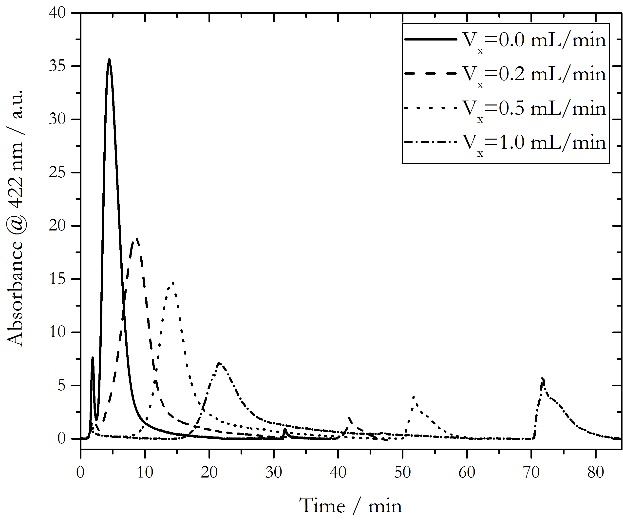 |
| --- | --- |
| (**a**) water pH 8 | (**b**) 0.05% v/v Mucasol |
| 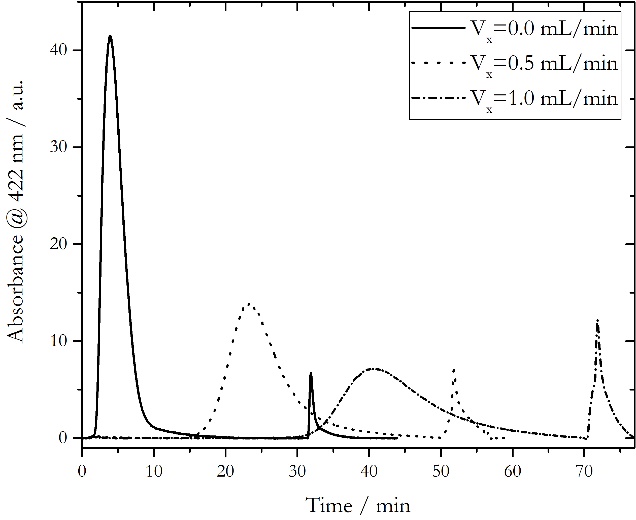 | |
| (**c**) 1 mM NaCl | |

Fig. S3 Fractograms of 50 nm AgNP_LA for different applied cross-flows and different carrier solutions.

| 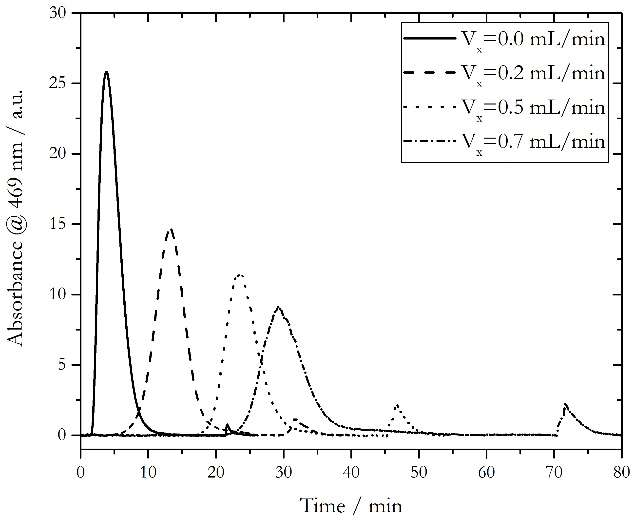 | 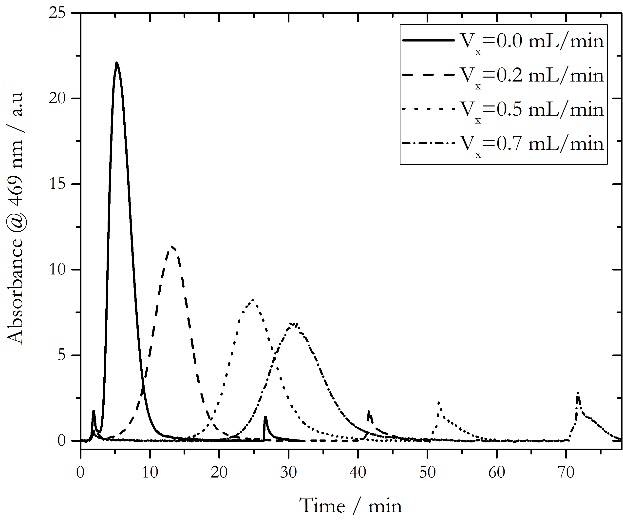 |
| --- | --- |
| (**a**) water pH 8 | (**b**) 0.05% v/v Mucasol |
| 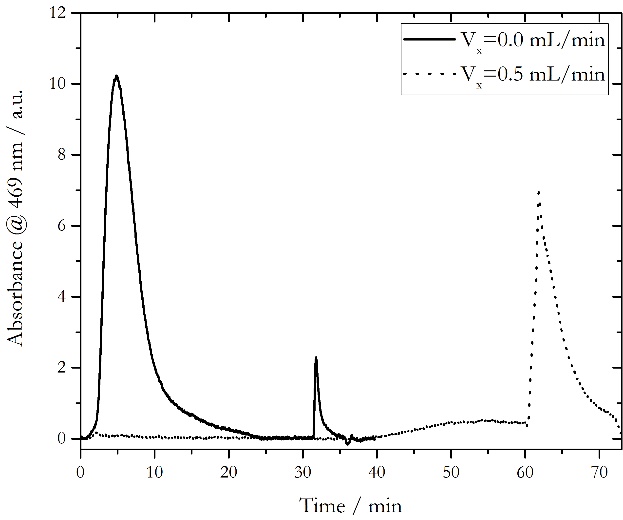 | |
| (**c**) 1 mM NaCl | |

Fig. S4 Fractograms of 80 nm AgNP_LA for different applied cross-flows and different carrier solutions.

**4. Retention times for 50 nm and 80 nm AgNP_LA**

| 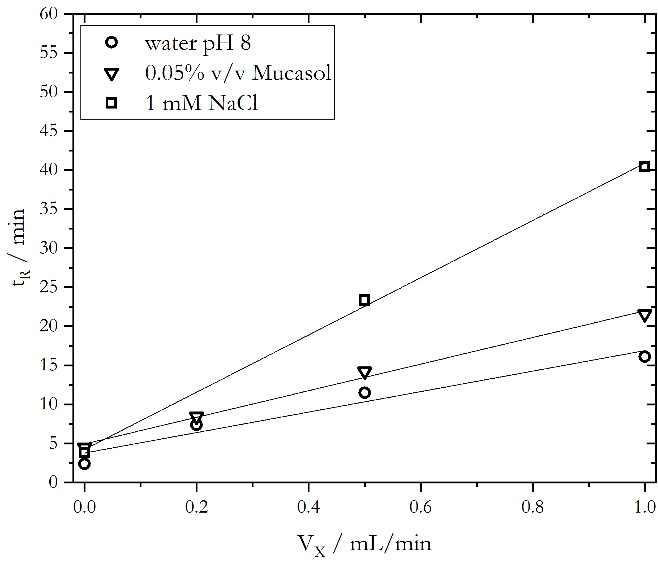 | 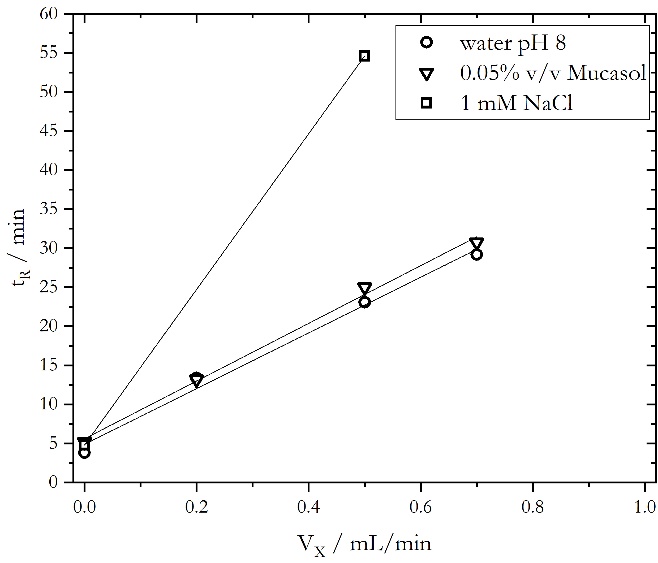 |
| --- | --- |
| (**a**) 50 nm AgNP_LA | (**b**) 80 nm AgNP_LA |

Fig. S5 Effect of carrier solution and applied cross-flow *V_x_* on retention time *t_R_.*

**5. Aggregation of AgNP_LA in fjord water**

| 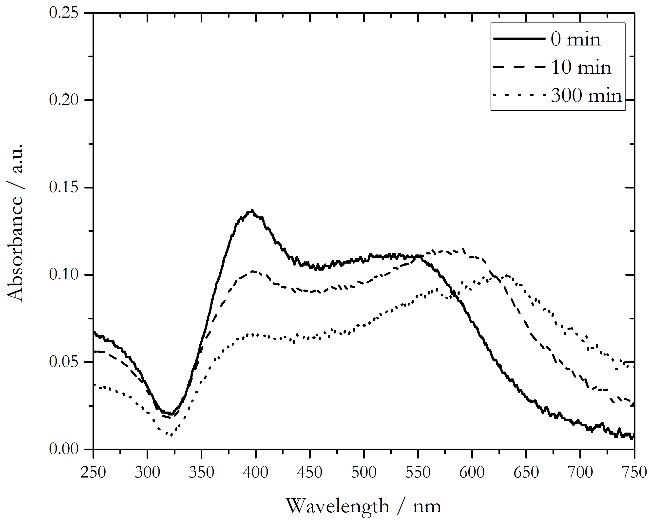 | 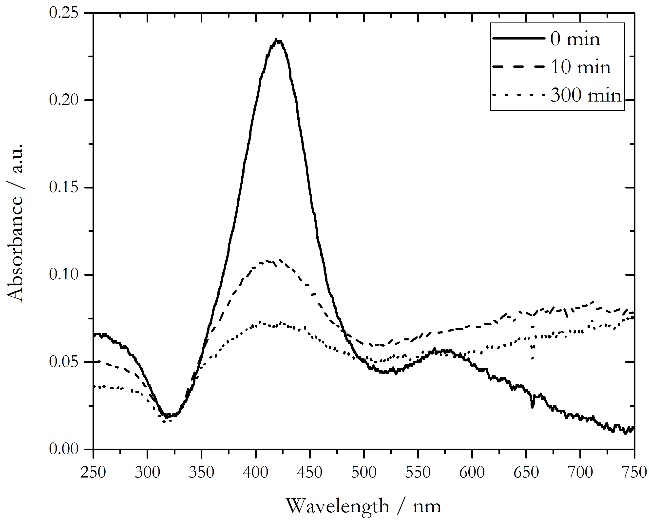 |
| --- | --- |
| (**a**) 20 nm AgNP_LA | (**b**) 50 nm AgNP_LA |
| 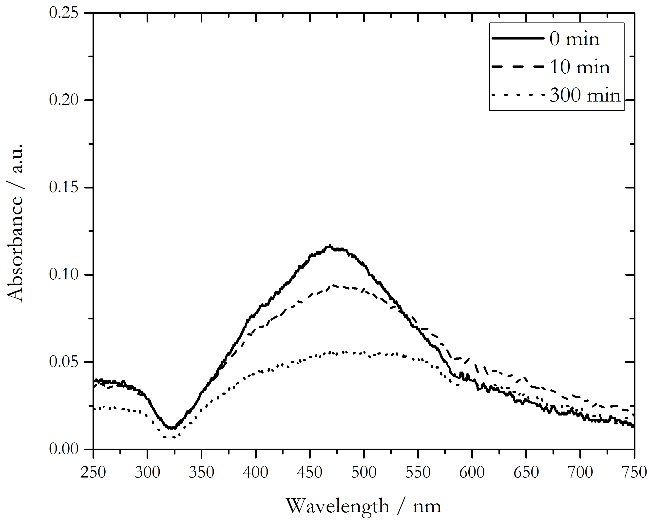 | 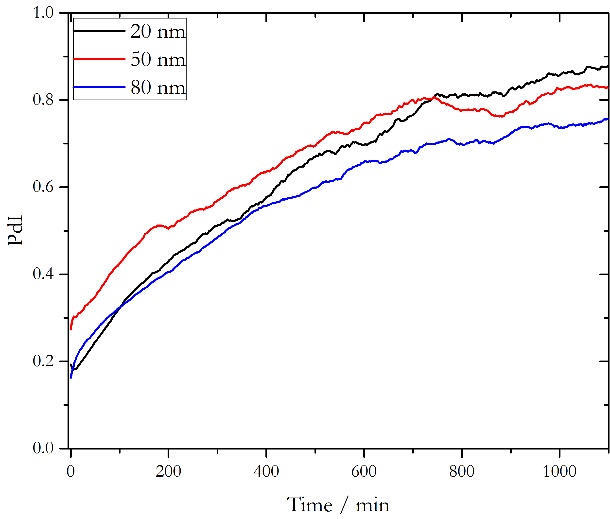 |
| (**c**) 80 nm AgNP_LA | (**d**) temporal trend of *PdI* |

Fig. S6 (a, b, c) UV/Vis spectra of all three AgNP_LA size fractions acquired at 0, 10, and 300 minutes after spiking into fjord water and (d) temporal trend of polydispersity index of AgNP_LA after spiking into fjord water.

**6. Size calibration**

Based on the previous findings, AF4 can be a powerful tool to evaluate particles’ hydrodynamic diameters (*d_H_)* of a sample with unknown particles size based on their individual *t_R_*. For this purpose, size calibration (*t_R_* vs. *d_H_*) has to be conducted for each individual type of nanoparticles, carrier solutions, membranes and applied cross-flows. Fig. S7 shows size calibrations for AgNP_LA at different cross-flows and carrier solutions.


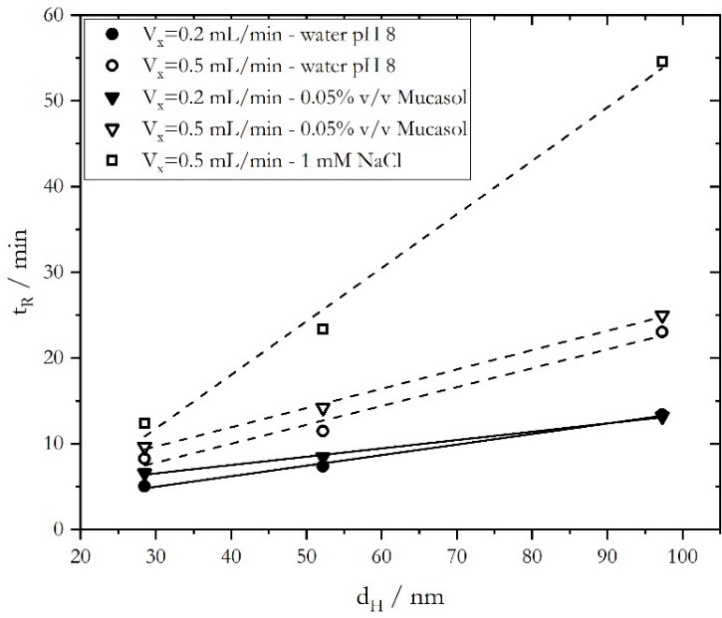


Fig. S7 Size calibration of the used AF4 system for two different cross-flows (*V_x_* = 0.2 mL/min: closed symbols; *V_x_* = 0.5 mL/min: open symbols) and different carrier solutions. Hydrodynamic diameters were taken from the DLS characterization (Table 2).

Table S2. Linear fits of *t_R_* vs *d_H_* plots from Fig. S7.

| **Carrier solution** | ***V_x_*** | **Linear fit** | **R²** |
| --- | --- | --- | --- |
| Water pH 8 | 0.2 mL/min  0.5 mL/min | *t_R_* = 0.123 min∙nm^-1^ *d_H_* + 1.309 min  *t_R_* = 0.220 min∙nm^-1^ *d_H_* + 1.215 min | 0.990  0.963 |
| 0.05% v/v Mucasol | 0.2 mL/min  0.5 mL/min | *t_R_* = 0.097 min∙nm^-1^ *d_H_* + 3.649 min  *t_R_* = 0.225 min∙nm^-1^ *d_H_* + 2.939 min | 0.990  0.995 |
| 1 mM NaCl | 0.5 mL/min | *t_R_* = 0.624 min∙nm^-1^ *d_H_* – 6.915 min | 0.983 |

The slope of the obtained linear fits increased with a larger applied cross-flow (Table S2 and solid lines vs. dashed lines in Fig. S7), which implies a better separation for samples containing different sized nanoparticles. The biggest gradient, so the maximum delayed elution time between different size fractions, was achieved with the use of 1 mM NaCl as carrier solution. However, a negative intercept was obtained for this calibration. One can assume that this is the result of a deviation from the linear behavior due to enhanced particle-membrane interactions at high cross flows and 1 mM NaCl carrier solution as the size of the AgNP_LA increased. Deviations from linearity were also obtained in other studies (1,2). Moreover, the use of 1 mM NaCl has a negative effect on the duration of the separation experiment, with the elution of 80 nm AgNP_LA after 55 minutes *versus* 25 minutes when 0.05% v/v Mucasol was used. The anomalously long *t_R_* when using 1 mM NaCl as carrier solution, together with the low recoveries and the obtained AF4 fractogram (Fig. S5) showing a very sharp peak, indicate that the NPs were aggregated along the AF4 channel and then partially removed from the system as a release peak. Thus we selected the carrier solutions water pH 8 and 0.05% v/v Mucasol for the calibration approach.

**References**

1. Geiss O, Cascio C, Gilliland D, Franchini F, Barrero-Moreno J. Size and mass determination of silver nanoparticles in an aqueous matrix using asymmetric flow field flow fractionation coupled to inductively coupled plasma mass spectrometer and ultraviolet-visible detectors. J Chromatogr A. 2013;1321:100–8.

2. Gigault J, Pettibone JM, Schmitt C, Hackley VA. Rational strategy for characterization of nanoscale particles by asymmetric-flow field flow fractionation: A tutorial. Anal Chim Acta. 2014 Jan;809:9–24.
